# Trajectories of health conditions predict cardiovascular disease risk among middle-aged and older adults: a national cohort study

**DOI:** 10.3389/fnut.2025.1657587

**Published:** 2025-09-12

**Authors:** Wenlong Li, Tian Liu, Yuanjia Hu, Hanwen Zhou, Yingcheng Liu, Haijiao Zeng, Yuan Zhang, Cong Zhang, Kangjie Li, Zuhai Hu, Pinyi Chen, Hua Wang, Biao Xie, Xiaoni Zhong

**Affiliations:** ^1^Department of Health Statistics, School of Public Health, Chongqing Medical University, Chongqing, China; ^2^College of Science, Xichang University, Xichang, China

**Keywords:** trajectories of health conditions, cardiovascular disease, latent class growth model, machine learning, SHapley Additive exPlanations

## Abstract

**Background:**

Most previous studies have focused on the association between health conditions measured at a single time point and the risk of cardiovascular disease (CVD), while evidence regarding the impact of long-term trajectories of health conditions is limited. This study aimed to construct models of health condition trajectories and to evaluate their association with CVD risk and predictive value.

**Methods:**

This study included 2,512 participants aged 45 years and older from the China Health and Retirement Longitudinal Study (CHARLS), who were followed from 2011 to 2018. Trajectories of multimorbidity status, activities of daily living (ADLs) limitations, body roundness index (BRI), pain, sleep duration, depressive symptoms, and cognitive function were identified using latent class growth models (LCGMs). Cox regression models were used to assess associations between these trajectories and incident CVD. Ten machine learning (ML) algorithms were applied to evaluate the predictive capacity of different variable groups for CVD. Additionally, SHapley Additive exPlanations (SHAP) values were used to interpret predictor importance and direction in the machine learning models.

**Results:**

Distinct high-risk trajectories of physical and psychological health were independently associated with increased CVD risk. Higher risks of CVD were observed for the moderate-ascending (HR = 1.42, 95% CI: 1.08–1.89) and high-ascending (3.01, 2.16–4.20) trajectories of multimorbidity status; the high-ascending trajectory of ADLs limitations (2.58, 1.87–3.56); the high-stable trajectory of BRI (1.67, 1.03–2.70); the moderate-ascending (1.51, 1.07–2.12) and high-ascending (2.28, 1.56–3.35) trajectories of pain; the moderate-descending (1.51, 1.09–2.10), low-ascending (1.70, 1.22–2.38), and high-posterior-ascending (2.54, 1.69–3.82) trajectories of depressive symptoms; and the low-ascending trajectory of sleep duration (1.33, 1.02–1.74). Notably, the model based on trajectories of health conditions achieved the highest predictive performance among all variable groups (CatBoost AUC = 0.740), with SHAP analysis confirming that the trajectories of multimorbidity status, BRI, and ADLs limitations were the most influential predictors.

**Conclusion:**

Long-term deterioration in both physical and psychological health is strongly associated with increased CVD risk, highlighting the importance of early intervention and continuous health monitoring.

## Introduction

Cardiovascular disease (CVD) remains the leading cause of death and disability worldwide (1). With the aging of the population and the increasing coexistence of physical and psychological health problems, the prevention and management of CVD are becoming increasingly complex, especially among middle-aged and older adults (2). In China, rapid urbanization and population aging have further aggravated the social and economic burden caused by CVD (3, 4). Therefore, early identification of high-risk individuals is crucial for preventing CVD events and reducing the disease burden.

A large number of studies have confirmed that impairments in physical and psychological health are closely associated with increased risk of CVD (5–11). However, most studies rely on cross-sectional assessments at a single time point and fail to reveal the dynamic trajectories of health status over time (12, 13), which may underestimate the true relationship between health changes and CVD risk. Recent studies have shown that analyzing health trajectories based on longitudinal data can better reflect population heterogeneity and improve the prediction of CVD risk (14). Existing studies have found that adverse trajectories of health conditions such as body roundness index (BRI) (15), sleep duration (9), and depressive symptoms (16) are significantly associated with increased CVD risk.

Most existing studies have focused only on a single domain of either physical or psychological health and lack comprehensive analyses integrating multiple health trajectories; although traditional biomarkers can be used for CVD risk assessment (17, 18), they fail to reflect the dynamic and multidimensional changes in health status. Integrating multiple health trajectories may provide a more comprehensive and dynamic understanding of the mechanisms underlying CVD and improve its risk prediction.

To address these gaps, we aimed to (1) identify distinct trajectories of multimorbidity status, limitations in activities of daily living (ADLs), BRI, pain, sleep duration, depressive symptoms, and cognitive function using latent class growth models (LCGMs) based on longitudinal data from the China Health and Retirement Longitudinal Study (CHARLS); (2) evaluate their associations with incident CVD; and (3) assess the incremental predictive value of these trajectories for CVD risk using machine learning (ML) approaches, with SHapley Additive exPlanations (SHAP) applied to interpret the contributions and directions of key predictors. These findings may help to improve precise risk stratification and early intervention for CVD, and promote individualized as well as community-level disease prevention strategies.

## Methods

### Study population

CHARLS is a nationally representative longitudinal cohort of Chinese residents aged 45 years and older, initiated by Peking University in 2011 to collect high-quality microdata for research on aging-related issues. The baseline survey, conducted from June 2011 to March 2012, included 17,708 individuals from 10,257 households across 150 counties or districts and 450 villages or urban communities in 28 provinces. Follow-up waves were conducted biennially: Wave 2 (2013–2014), Wave 3 (2015–2016), and Wave 4 (2017–2018). Data collection used face-to-face computer-assisted personal interviews (CAPI) and included comprehensive assessments of demographic characteristics, socioeconomic status, health status, physical measurements, and biomarkers. To ensure data quality, CHARLS employed rigorous quality control procedures at each wave, including standardized interviewer training, centralized field supervision, built-in logic and range checks within the CAPI system, and double data entry for verification. The study protocol was approved by the Institutional Review Board of Peking University (IRB00001052-11015) and adhered to the STROBE guidelines (19).

**Figure 1:**
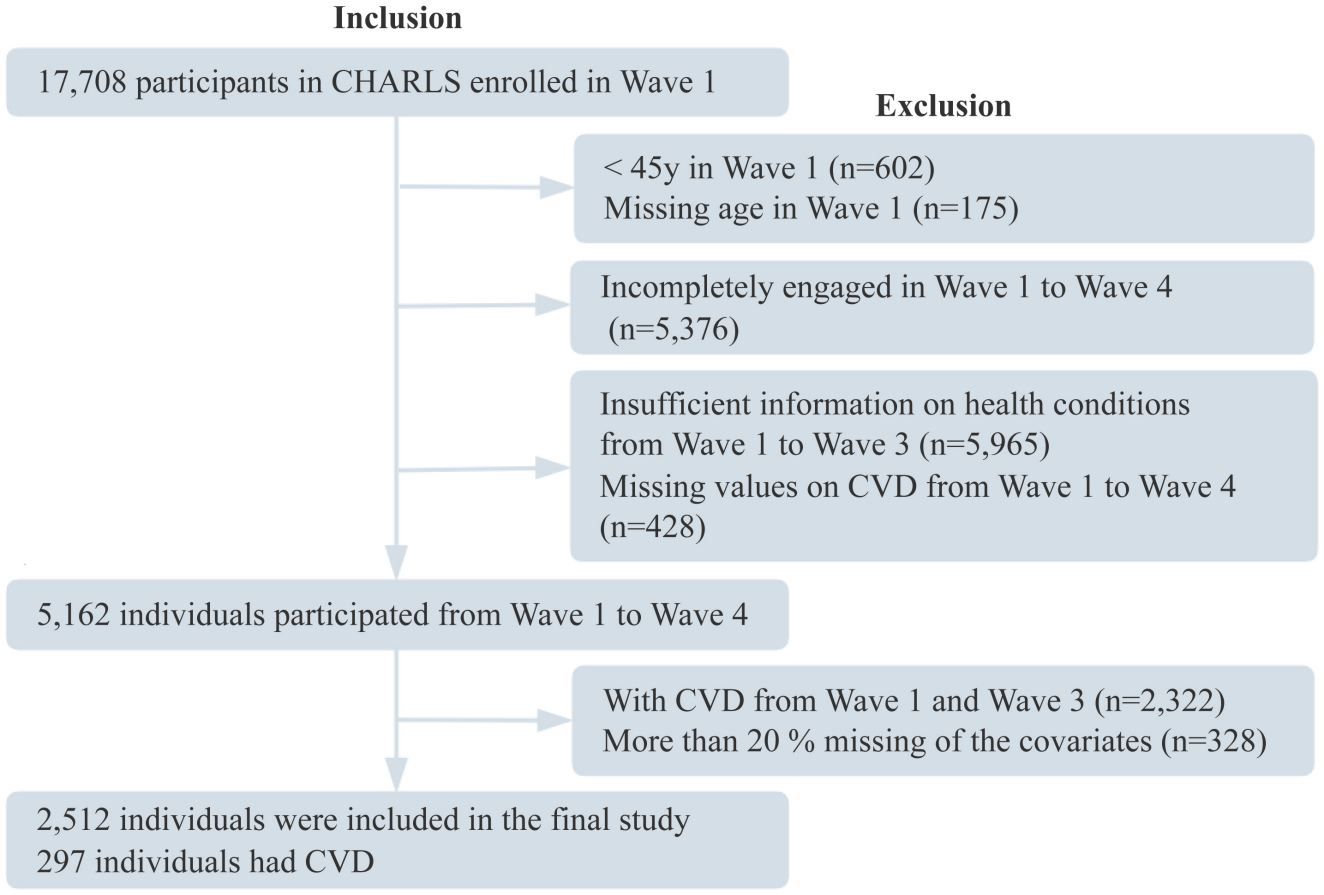
Participant flowchart. CHARLS, China Health and Retirement Longitudinal Study; CVD, cardiovascular disease.

In this study, physical and psychological data from Waves 1, 2, and 3 were used to identify trajectories of multimorbidity status, ADLs limitations, BRI, pain, sleep duration, depressive symptoms, and cognitive function. Data from Wave 4 were analyzed to examine the associations between these trajectories and CVD incidence. Exclusion criteria were as follows: (1) age < 45 years in Wave 1; (2) incomplete follow-up from Wave 1 to Wave 4; (3) missing health condition data in any of Waves 1–3; (4) incomplete CVD data across Waves 1–4; (5) confirmed diagnosis of CVD in Waves 1–3; and (6) missing data in ≥20% of covariates. The final analytic sample included 2,512 participants. The flow of participant selection is shown in Figure 1.

### Assessment of health conditions

Multimorbidity status was assessed based on self-reported physician diagnoses of 12 chronic conditions, including hypertension, diabetes, dyslipidemia, chronic lung disease, asthma, kidney disease, liver disease, gastrointestinal disorders, cancer, psychiatric disorders, memory-related conditions, and arthritis. Multimorbidity was defined as the presence of two or more chronic conditions in the same individual, has been widely employed in large-scale studies of Chinese middle-aged and older adults (20, 21). Each condition was assigned a score of 1, with a maximum possible score of 12, where higher scores indicate greater severity of multimorbidity.

ADLs limitations are assessed through a comprehensive evaluation of basic activities of daily living (BADLs) and instrumental activities of daily living (IADLs) (22). BADLs include six items: dressing, bathing, eating, transferring in and out of bed, toileting, and managing urinary and bowel function. IADLs comprise five items: performing household chores, meal preparation, shopping, managing finances, and medication adherence. Each item is rated on a four-point scale: (1) no difficulty; (2) difficulty but manageable; (3) difficulty requiring assistance; and (4) inability to perform. This assessment focuses on long-term physical function, excluding difficulties expected to resolve within 3 months. A score of 0 is assigned to items with no difficulty, while any degree of difficulty is scored as 1. The total ADLs limitations score is calculated by summing the scores for BADLs and IADLs. Participants who do not complete all items within either BADLs or IADLs are excluded from the calculation. The possible score ranges for IADLs, BADLs, and overall ADLs limitations are 0–5, 0–6, and 0–11, respectively; higher scores indicate greater limitations in physical activity. The ADL scale has been extensively applied in previous studies of older adults in China, demonstrating robust reliability and validity (23, 24).

BRI was calculated as $364.2-365.5\times \sqrt{1-\frac{{\left[\;waist\;circumference\;\left(cm\right)/2\pi \right]}^{2}}{{\left[0.5\times \;height\;\left(cm\right)\right]}^{2}}}$ (25, 26). Height was measured to the nearest 0.1 cm using a stadiometer with participants standing upright and barefoot. Waist circumference was measured to the nearest 0.1 cm at the level of the umbilicus at the end of normal expiration using a non-stretchable tape.

Pain was assessed by asking participants to report all areas of the body currently experiencing pain, including the head, shoulders, arms, chest, abdomen, back, waist, hips, legs, knees, ankles, and neck (27). The total number of painful sites was calculated, ranging from 0 to 15, with higher counts indicating greater pain severity. This approach has been widely used in large-scale epidemiological studies in Chinese adults (28, 29).

Nighttime sleep duration was assessed by asking, “During the past month, how many hours of actual sleep did you get at night?” Responses were recorded as integers. This question was adapted from the Pittsburgh Sleep Quality Index (PSQI) (30, 31), a validated instrument with established reliability and validity in prior research (32).

Depressive symptoms were assessed using the 10-item short form of the Center for Epidemiologic Studies Depression Scale (CESD-10), a validated self-report instrument (33) widely used in epidemiological surveys. Participants were asked to recall their feelings over the past week and rate 10 items (including being bothered by trivial matters, having difficulty concentrating, feeling depressed, feeling that everything was an effort, feeling hopeful, feeling fearful, experiencing restless sleep, feeling happy, feeling lonely, and feeling unable to continue) on a scale from 0 [rarely or none of the time (< 1 day)] to 3 [most or all of the time (5–7 days)]. Items 5 and 8 were reverse-scored before calculating the total score, which ranged from 0 to 30, with higher scores indicating more severe depressive symptoms. The CESD-10 has demonstrated good reliability and validity in older Chinese adults (34).

The cognitive assessment was adapted from the Health and Retirement Study (HRS). Consistent with previous studies (35, 36), cognitive function was categorized into two domains: episodic memory and mental intactness. Episodic memory was evaluated using the word recall test, which included both immediate and delayed recall of 10 words. Each task was scored from 0 to 10 points, yielding a total of 20 points. Mental intactness was assessed using items from the 10-item Telephone Interview for Cognitive Status (TICS-10), comprising serial subtraction of 7 from 100 up to five times (5 points), orientation to the current year, month, day, day of the week, and season (5 points), and reproduction of two overlapping pentagons (1 point). The global cognitive score was calculated as the sum of the episodic memory and mental intactness scores, ranging from 0 to 31, with higher scores indicating better cognitive function. This instrument has been shown to be a reliable and valid measure of cognitive function in Chinese middle-aged and older adults in prior research (37, 38).

### Assessment of CVD events

The primary outcome was incident CVD events ascertained in Wave 4, including heart disease and stroke. Consistent with previous studies (39–41), CVD events were identified based on self-reported physician-diagnosed in response to the questions: “Has a doctor ever diagnosed you with a heart attack, angina, coronary artery disease, heart failure, or other cardiovascular condition?” or “Has a doctor ever informed you that you have had a stroke?” Participants who reported a new diagnosis of heart disease or stroke during follow-up were considered to have incident CVD events. To ensure the accuracy of these self-reported outcomes, CHARLS implemented internal consistency checks during follow-up interviews: participants who had reported heart disease or stroke in the previous wave were asked to reconfirm the diagnosis, and if they denied the prior report, the original record was retrospectively corrected to reduce recall bias and enhance the validity of outcome ascertainment (42).

### Covariates

Covariates were assessed using data from the 2015 survey (Wave 3) of CHARLS.

Sociodemographic and lifestyle variables were obtained through structured, face-to-face interviews conducted by trained interviewers. Sociodemographic variables included age, sex, marital status (married vs. unmarried), residence (rural vs. urban), and educational level (primary school or below, middle school, high school or above). Lifestyle factors comprised smoking status (current, former, or never) and drinking status (drinking >1/week, ≤ 1/week, or never).

Physical measurements were obtained following standardized procedures (43). Blood pressure (BP) was measured with an electronic sphygmomanometer (HEM-7200 Monitor) after 5 min of rest in the sitting position, and the mean of three BP measurements was used in the analyses. WC was measured using nonstretched tape at the navel level at minimal respiration. Height was measured with a 213 stadiometer with participants standing upright and barefoot on the floor board of the instrument. Weight was measured using an HN-286 scale, and BMI was calculated as weight in kilograms divided by height squared (m^2^).

Laboratory measurements were based on fasting venous blood samples collected at township hospitals or community health centers using EDTA-K_2_ anticoagulant vacuum tubes. Samples were processed within 2 h of collection, with plasma and buffy coat separated by centrifugation, aliquoted into cryovials, and stored at −20°C on site. All specimens were transported on dry ice via a monitored cold chain (temperature recorded every 5 min) to KingMed Diagnostics (Tianjin, China), a College of American Pathologists–and ISO 15189–accredited laboratory (44), where assays were performed using standardized protocols with daily internal quality-control runs reviewed weekly by the CHARLS research team. Measured biomarkers included triglycerides (TG), creatinine (CREA), high-density lipoprotein cholesterol (HDL-C), low-density lipoprotein cholesterol (LDL-C), total cholesterol (TC), fasting blood glucose (GLU), uric acid (UA), and C-reactive protein (CRP). Details of assay methods, coefficients of variation, and detection limits are provided in Supplementary Table S1.

### Statistical analysis

Model fit and optimal latent class selection were evaluated using the Akaike Information Criterion (AIC), Bayesian Information Criterion (BIC), adjusted BIC (aBIC), Bootstrapped Likelihood Ratio Test (BLRT), Lo-Mendell-Rubin (LMR) test, and entropy. Statistically significant BLRT and LMR *P*-values (< 0.05) indicated that the k-class model provided a better fit than the (k-1)-class model. The optimal model was determined by lower AIC, BIC, and aBIC values and higher entropy. Each latent class was required to include at least 5% of the sample, and clarity and interpretability of trajectories were also considered. After trajectory identification, a nominal categorical variable was generated to assign participants to trajectory groups. Model fit and trajectory separation were further assessed by data visualization.

To evaluate the risk of CVD, the endpoint of Wave 3 was set as the baseline for survival analyses. The duration from the baseline (2015) to the occurrence of a CVD event, death, or loss to follow-up was recorded as the follow-up time. Cox proportional hazards models were used to estimate hazard ratios (HRs) and 95% confidence intervals (CIs) for the association between trajectories of health conditions and incident CVD. Three models were fitted: Model 1 included no covariates; Model 2 was adjusted for age and gender. To further address potential confounding by sociodemographic factors, physical examination, and blood test results, Model 3 additionally adjusted for marital status, residence, education level, drinking status, smoking status, BMI, SBP, DBP, TG, CREA, HDL-C, LDL-C, TC, GLU, UA, and CRP. Subgroup analyses and interaction tests were also conducted to examine whether associations between trajectories of health conditions and CVD risk differed by age, gender, marital status, education level, residence, smoking status, drinking status, BMI, SBP, and DBP.

ML comprises a variety of algorithms capable of revealing complex relationships among variables, making it an important tool for disease prediction. With advances in computational technology and the widespread application of large-scale datasets, the role of ML in health risk prediction has become increasingly prominent (45). In this study, the data were randomly divided into training and testing sets at a ratio of 8:2. Ten ML algorithms were used, including Logistic Regression (LR), Support Vector Machine (SVM), Gradient Boosting Machine (GBM), Neural Network (NN), Random Forest (RF), Extreme Gradient Boosting (XGBoost), K-Nearest Neighbors (KNN), AdaBoost, LightGBM, and CatBoost, to evaluate the predictive ability of four variable groups: sociodemographic factors, blood tests, physical examinations, and trajectories of health conditions for CVD risk. To systematically compare the predictive contributions of each variable group, two modeling strategies were adopted. First, in the model including all variable groups, each group was sequentially excluded to assess its impact on model performance. Second, independent models were constructed by including only one variable group to evaluate its individual predictive ability. Model performance was evaluated using the area under the receiver operating characteristic curve (AUC) to quantify the contribution of each variable group to CVD risk prediction. In addition, to enhance model interpretability, SHAP analysis was conducted on the optimal ML model based on trajectories of health conditions, and a summary plot was used to visualize the importance of each feature and its impact direction on the model output.

Outliers in continuous variables were defined as values more than 1.5 times the interquartile range (IQR) below the first quartile (Q1) or above the third quartile (Q3) and were excluded from the analysis. Covariates with less than 20% missingness were subsequently imputed using the missForest algorithm, a nonparametric random forest–based approach capable of handling both continuous and categorical variables by leveraging observed values from other features to predict missing ones (46). For continuous variables with a normal distribution, data are presented as mean ± standard deviation (SD) and compared using analysis of variance (ANOVA), whereas variables with a non-normal distribution are presented as median (IQR) and compared using the Kruskal–Wallis H test. LCGM analyses were conducted using Mplus version 8.3, and all other analyses were performed using R version 4.4.2 and Python version 3.11.7. A two-sided *p*-value < 0.05 was considered statistically significant.

## Results

### Identification of trajectories of health conditions

**Figure 2:**
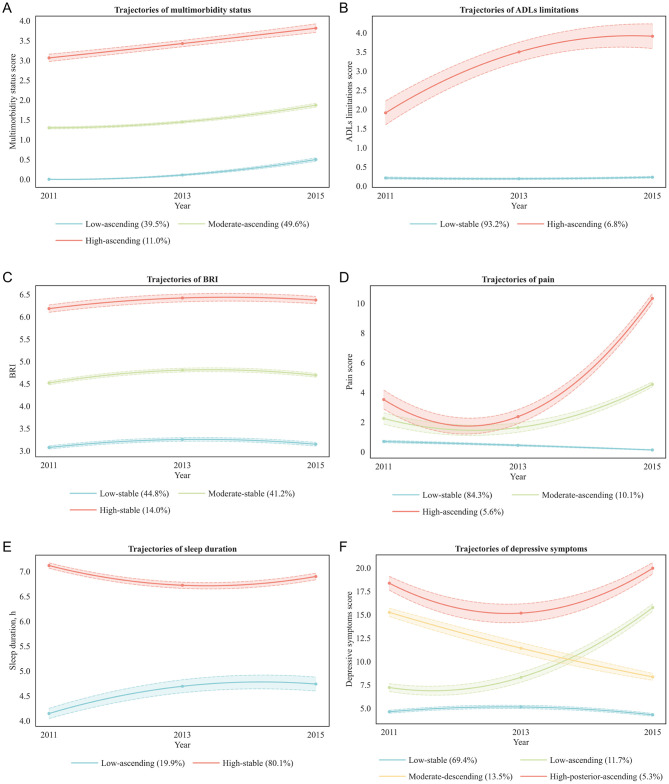
Trajectories of health conditions. BRI, body roundness index; ADLs, activities of daily living. **(A)** Multimorbidity status: low-ascending (blue line, low baseline multimorbidity scores with gradual increase; 39.5% of participants), Moderate-ascending (green line, moderate baseline scores with steady increase; 49.6%), High-ascending (red line, high baseline scores with further increase; 11.0%). **(B)** ADLs limitations: low-stable (blue line, persistently low limitation scores; 93.2%), High-ascending (red line, high baseline scores with progressive increase; 6.8%). **(C)** BRI: low-stable (blue line, low baseline BRI maintained over time; 44.8%), moderate-stable (green line, moderate baseline BRI remaining stable; 41.2%), high-stable (red line, high baseline BRI maintained over time; 14.0%). **(D)** Pain: low-stable (blue line, persistently low pain scores; 84.3%), moderate-ascending (green line, moderate baseline scores with steady increase; 10.1%), high-ascending (red line, high baseline scores with marked increase; 5.6%). **(E)** Sleep duration: low-ascending (blue line, short baseline sleep duration with gradual increase; 19.9%), high-stable (red line, long baseline sleep duration maintained over time; 80.1%). **(F)** Depressive symptoms: low-stable (blue line, low baseline depressive symptom scores maintained over time; 69.4%), low-ascending (green line, low baseline scores with gradual increase; 11.7%), moderate-descending (orange line, moderate baseline scores with gradual decrease; 13.5%), High-posterior-ascending (red line, high baseline scores with early decline followed by later increase; 5.3%).

To provide a more comprehensive understanding of the longitudinal changes in health conditions among middle-aged and older adults, this study applied LCGM to analyze the trajectories of multimorbidity status, ADLs limitations, BRI, pain, sleep duration, depressive symptoms, and cognitive function. The study population was divided into five categories (CLASS-1 to CLASS-5), and the optimal model for each health condition was selected based on evaluation metrics including AIC, BIC, aBIC, entropy, LMR (*P*), and BLRT (*P*) (Supplementary Tables S2–S8). Ultimately, the optimal trajectories for the seven health conditions were determined (Figure 2; Supplementary Figure S1).

The trajectories of multimorbidity status were best represented by a quadratic estimation class-3 model (AIC = 16,380.4; BIC = 16,467.9; aBIC = 16,420.2; entropy = 0.940). Under the same number of classes, quadratic estimation yielded lower AIC, BIC, and aBIC than linear and free estimation. The class-3 model fit significantly better than the class-2 model (BLRT and LMR, both *P* < 0.001), whereas the class-4 model offered no additional improvement (LMR *P* = 0.237), indicating that three distinct and well-separated classes most appropriately described the data.

The trajectories of ADLs limitations were best captured by a free estimation class-2 model (AIC = 18,391.5; BIC = 18,444.0; aBIC = 18,415.4; entropy = 0.988). Although quadratic estimation achieved the lowest information criteria overall, its class-2 model did not pass the LMR test (*P* = 0.061), and its class-3 model contained classes with proportions below 5% (0.079/0.025/0.025), limiting clinical interpretability. Free estimation outperformed linear estimation in all information criteria, and the class-2 free estimation model fit significantly better than the class-1 model (BLRT and LMR, both *P* < 0.001), whereas the class-3 model was not significant (LMR *P* = 0.072). This suggests that a simpler two-class solution offered both statistical robustness and practical interpretability.

The trajectories of BRI were best represented by a free estimation class-3 model (AIC = 20,429.7; BIC = 20,499.6; aBIC = 20,461.5; entropy = 0.851). Although quadratic estimation produced the lowest overall information criteria, its entropy was markedly lower than that of linear and free estimation. Free estimation yielded lower information criteria than linear estimation, and the class-3 model showed significant improvement over the class-2 model (BLRT and LMR, both *P* < 0.001) with all class proportions ≥5%, supporting its selection as the optimal balance between model fit and interpretability.

The trajectories of pain were best captured by a free estimation class-3 model (AIC = 28,632.0; BIC = 28,702.0; aBIC = 28,663.9; entropy = 0.988). Although quadratic estimation yielded lower overall information criteria, its class-3 model contained a small class (0.083/0.030/0.887), limiting interpretability. Free estimation outperformed linear estimation, and the class-3 model significantly improved fit over the class-2 model (BLRT and LMR, both *P* < 0.001) with all class proportions ≥5%, ensuring stable classification accuracy.

The trajectories of sleep duration were best represented by a quadratic estimation class-2 model (AIC = 28,197.2; BIC = 28,261.3; aBIC = 28,226.4; entropy = 0.698). Quadratic estimation had the lowest overall information criteria, and the class-2 model fit significantly better than the class-1 model (BLRT and LMR, both *P* < 0.001) with all class proportions ≥5%. The class-3 model showed further decreases in information criteria but reduced entropy (0.580), suggesting potential overfitting without substantive improvement in interpretability.

The trajectories of depressive symptoms were best captured by a quadratic estimation class-4 model (AIC = 44,872.98; BIC = 44,983.72; aBIC = 44,923.36; entropy = 0.825). Quadratic estimation produced the lowest information criteria overall, and the class-4 model showed further decreases compared with the class-3 model (AIC = 45,161.93; BIC = 45,249.36; aBIC = 45,201.70) with significant BLRT and LMR tests (both *P* < 0.001) and all class proportions ≥5%. The class-5 model yielded slightly lower information criteria but nonsignificant LMR (*P* = 0.150), confirming that the four-class model achieved the best trade-off between fit, complexity, and interpretability.

The trajectories of cognitive function were best represented by a free estimation class-2 model (AIC = 41,711.24; BIC = 41,763.70; aBIC = 41,735.10; entropy = 0.708). Although quadratic estimation showed the lowest overall information criteria, its entropy was low (0.626 for class-2, 0.615 for class-3), limiting stability. Free estimation yielded lower information criteria than linear estimation, and the class-2 model showed higher entropy than the class-3 model and significantly improved fit over the single-class model (BLRT and LMR, both *P* < 0.001) with all class proportions ≥5%, supporting the adoption of a parsimonious two-class solution.

### Baseline characteristics by trajectories of health conditions

**Figure 3:**
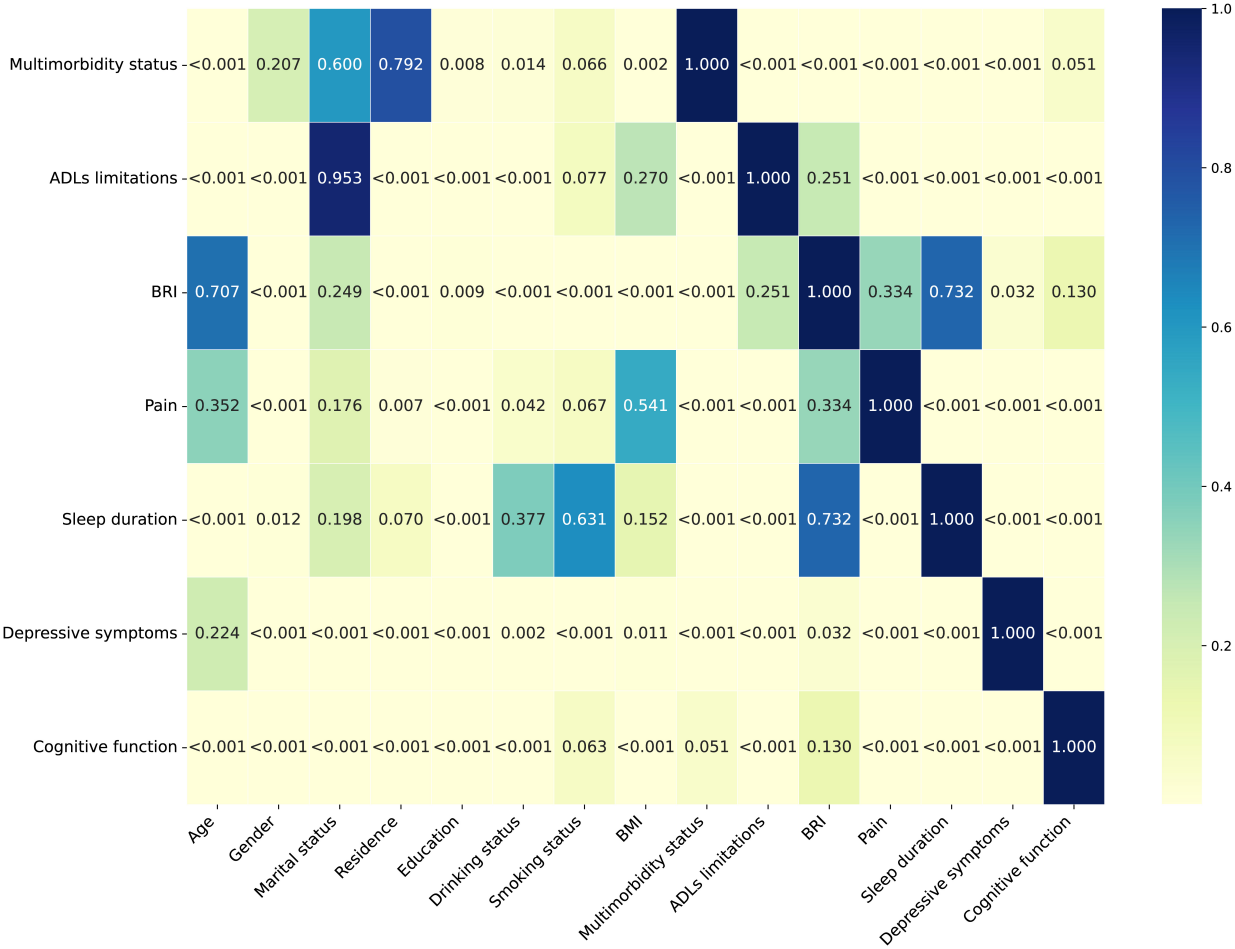
Heatmap of *P*-values for participants' characteristics stratified by trajectories of health conditions. BRI, body roundness index; ADLs, activities of daily living.

As shown in Figure 3, a heatmap of *p*-values revealed statistically significant differences (*P* < 0.05) in demographic and health-related characteristics across the different health condition trajectory groups. Specifically, individuals in the adverse health conditions trajectory group were older, had lower educational attainment, were more likely to live in rural areas, and had a higher proportion of females. In addition, the proportions of individuals who did not drink alcohol or smoke were also higher in this group. In terms of health, these individuals had more severe multimorbidity status, higher levels of ADLs limitations, BRI, pain, and depressive symptoms, shorter sleep duration, and poorer cognitive function (Supplementary Tables S9–S15).

### Baseline characteristics by incident CVD status

A total of 2,512 participants were included in the final analysis. Baseline characteristics are presented in Table 1. The mean age was 60.78 ± 7.85 years; 54.62% were male, 84.28% were married, and 65.64% resided in rural areas. During the follow-up period, 297 participants developed CVD. Participants who developed CVD were more likely to be older, smoke less, have lower education level, and have higher BMI, SBP, DBP, TG, and CRP. They were also more likely to have multimorbidity, ADLs limitations, higher BRI, greater pain, and more severe depressive symptoms.

**Table 1:** Baseline characteristics of participants.

**Characteristics**	**Total**	**No CVD**	**CVD**	***P***
*N*	2,512	2,215	297	
**Sociodemographic factors**				
Age	60.78 ± 7.85	60.53 ± 7.83	62.66 ± 7.78	<0.001
Gender				0.129
Female	1,140 (45.38)	993 (44.83)	147 (49.49)	
Male	1,372 (54.62)	1,222 (55.17)	150 (50.51)	
Marital status				0.530
Married	2,117 (84.28)	1,863 (84.11)	254 (85.52)	
Unmarried	395 (15.72)	352 (15.89)	43 (14.48)	
Residence				0.900
Rural	1,649 (65.64)	1,455 (65.69)	194 (65.32)	
Urban	863 (34.36)	760 (34.31)	103 (34.68)	
Education level				0.014
Primary school or lower	1,491 (59.36)	1,298 (58.60)	193 (64.98)	
Middle school	685 (27.27)	625 (28.22)	60 (20.20)	
High school or above	336 (13.38)	292 (13.18)	44 (14.81)	
Drinking status				0.238
Never drinking	1,543 (61.43)	1,348 (60.86)	195 (65.66)	
Drinking ≤ 1/week	448 (17.83)	398 (17.97)	50 (16.84)	
Drinking >1/week	521 (20.74)	469 (21.17)	52 (17.51)	
Smoking status				0.006
Never smoking	1,299 (51.71)	1,139 (51.42)	160 (53.87)	
Former smoking	427 (17.00)	362 (16.34)	65 (21.89)	
Current smoking	786 (31.29)	714 (32.23)	72 (24.24)	
**Physical examination factors**				
BMI, kg/m^2^	23.65 ± 3.43	23.55 ± 3.40	24.43 ± 3.55	<0.001
SBP, mmHg	127.96 ± 20.14	127.15 ± 19.84	134.06 ± 21.30	<0.001
DBP, mmHg	75.59 ± 11.82	75.38 ± 11.87	77.13 ± 11.31	0.016
**Blood test factors**				
TG, mmol/L	112.39 (82.30, 168.36)	111.50 (81.42, 167.26)	120.35 (89.38, 177.88)	0.023
CREA, μmol/L	0.81 ± 0.23	0.81 ± 0.23	0.81 ± 0.23	0.916
HDL-C, mmol/L	51.60 ± 11.89	51.64 ± 11.80	51.27 ± 12.63	0.618
LDL-C, mmol/L	103.11 ± 28.44	102.82 ± 28.08	105.22 ± 30.99	0.174
TC, mmol/L	185.04 ± 35.99	184.60 ± 35.88	188.27 ± 36.75	0.099
GLU, mmol/L	102.06 ± 30.78	101.67 ± 30.58	104.97 ± 32.08	0.083
UA, μmol/L	4.99 ± 1.39	4.98 ± 1.40	5.07 ± 1.29	0.262
CRP, mg/L	1.40 (0.80, 2.50)	1.40 (0.70, 2.40)	1.60 (0.90, 3.00)	<0.001
**Trajectories of health conditions factors**				
Multimorbidity status				<0.001
Low-ascending	991 (39.45)	915 (41.31)	76 (25.59)	
Moderate-ascending	1,245 (49.56)	1,099 (49.62)	146 (49.16)	
High-ascending	276 (10.99)	201 (9.07)	75 (25.25)	
ADLs limitations				<0.001
Low-stable	2,342 (93.23)	2,094 (94.54)	248 (83.50)	
High-ascending	170 (6.77)	121 (5.46)	49 (16.50)	
BRI				<0.001
Low-stable	1,126 (44.82)	1,028 (46.41)	98 (33.00)	
Moderate-stable	1,035 (41.20)	905 (40.86)	130 (43.77)	
High-stable	351 (13.97)	282 (12.73)	69 (23.23)	
Pain				<0.001
Low-stable	2,118 (84.32)	1,893 (85.46)	225 (75.76)	
Moderate-ascending	253 (10.07)	213 (9.62)	40 (13.47)	
High-ascending	141 (5.61)	109 (4.92)	32 (10.77)	
Sleep duration				0.032
High-stable	2,012 (80.10)	1,788 (80.72)	224 (75.42)	
Low-ascending	500 (19.90)	427 (19.28)	73 (24.58)	
Depressive symptoms				<0.001
Low-stable	1,744 (69.43)	1,570 (70.88)	174 (58.59)	
Moderate-descending	340 (13.54)	291 (13.14)	49 (16.50)	
Low-ascending	294 (11.70)	249 (11.24)	45 (15.15)	
High-posterior-ascending	134 (5.33)	105 (4.74)	29 (9.76)	
Cognitive function				0.020
High-stable	1,648 (65.61)	1,471 (66.41)	177 (59.60)	
Low-descending	864 (34.39)	744 (33.59)	120 (40.40)	

Data are presented as the mean (± standard deviation), median (interquartile range), or frequency (percentage), as appropriate. BMI, body mass index; SBP, systolic blood pressure; DBP, diastolic blood pressure; TG, triglycerides; CREA, creatinine; HDL-C, high-density lipoprotein cholesterol; LDL-C, low-density lipoprotein cholesterol; TC, total cholesterol; GLU, glucose; UA, uric acid; CRP, c-reactive protein; BRI, body roundness index; ADLs, activities of daily living; CVD, cardiovascular disease.

### Associations between trajectories of health conditions and CVD risk

**Figure 4:**
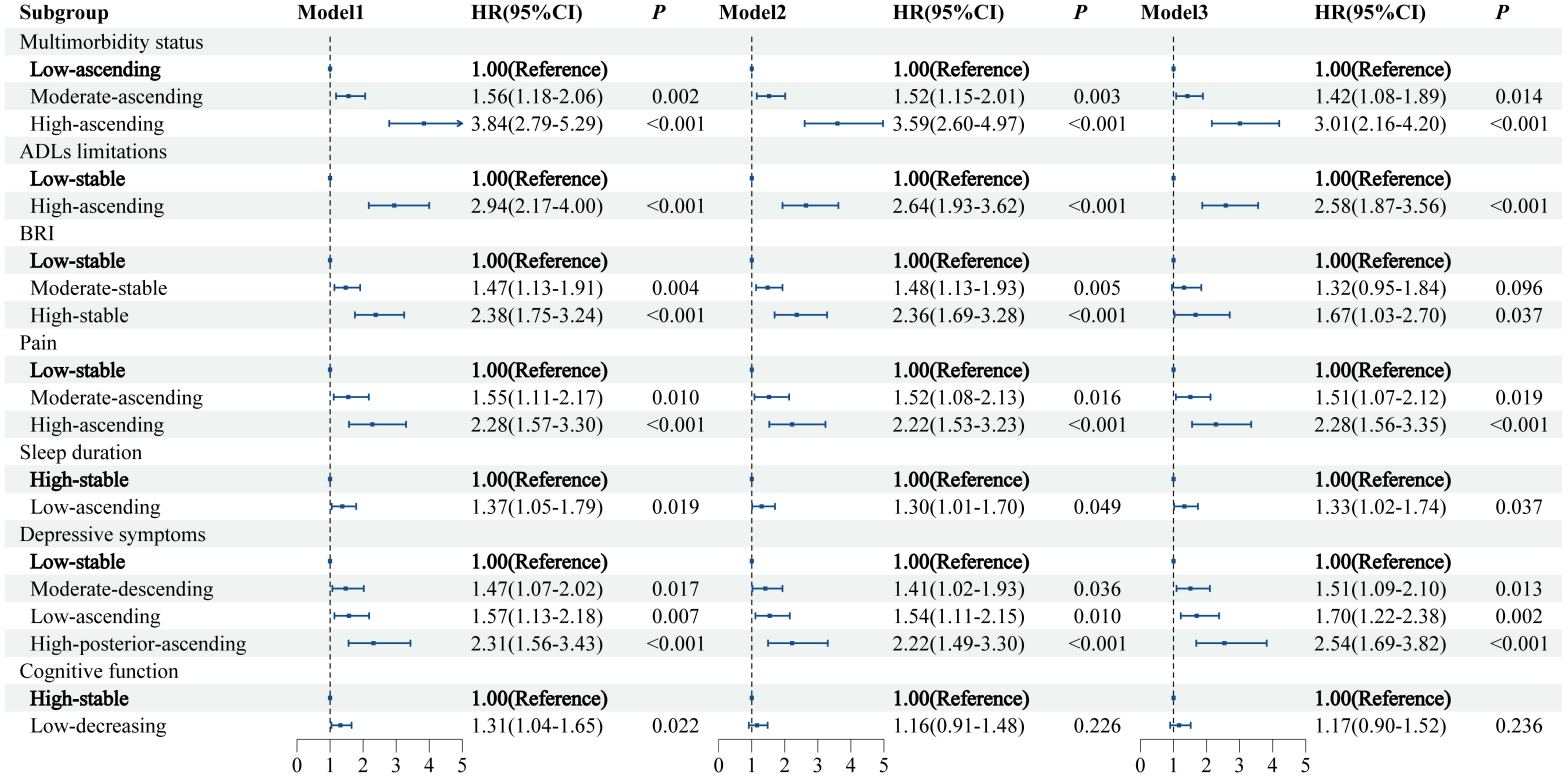
The relationship between trajectories of health condition and CVD risk. Model 1 was a univariate model. Model 2 was adjusted for age and gender. Model 3 was based on model 2, with additional adjustments for marital status, residence, education level, drinking status, smoking status, body mass index, systolic blood pressure, diastolic blood pressure, triglycerides, creatinine, high-density lipoprotein, low-density lipoprotein, total cholesterol, glucose, uric acid, and C-reactive protein. CVD, cardiovascular disease; BRI, body roundness index; ADLs, activities of daily living.

In the fully adjusted model (Model 3; Figure 4), we observed that several trajectories of health conditions were associated with an increased risk of CVD, as follows: the moderate-ascending (HR = 1.42, 95% CI: 1.08–1.89) and high-ascending (3.01, 2.16–4.20) trajectories of multimorbidity status (vs. low-ascending); the high-ascending trajectory of ADLs limitations (2.58, 1.87–3.56; vs. low-stable); the high-stable trajectory of BRI (1.67, 1.03–2.70; vs. low-stable); the moderate-ascending (1.51, 1.07–2.12) and high-ascending (2.28, 1.56–3.35) trajectories of pain (vs. low-stable); the moderate-descending (1.51, 1.09–2.10), low-ascending (1.70, 1.22–2.38), and high-posterior-ascending (2.54, 1.69–3.82) trajectories of depressive symptoms (all vs. low-stable); and the low-ascending trajectory of sleep duration (1.33, 1.02–1.74; vs. high-stable).

These associations remained robust in both unadjusted and age- and sex-adjusted models, except that the association between cognitive function trajectory and CVD risk was no longer significant after adjustment for age and sex.

### Subgroup analysis

We further explored these associations in prespecified subgroups (Supplementary Tables S16–S22). The high-ascending trajectory of ADLs limitations was associated with a substantially increased risk of CVD among participants with diastolic blood pressure ≥ 80 mmHg (4.27, 2.68–6.79; *P* for interaction = 0.003; Supplementary Table S17). The high-ascending trajectory of pain was associated with a higher risk of CVD among current smokers (5.56, 2.69–11.51; *P* for interaction = 0.01; Supplementary Table S19). For sleep duration, the low-ascending trajectory was associated with a higher risk of CVD among unmarried participants (2.68, 1.38–5.23; *P* for interaction = 0.044; Supplementary Table S20). Among women, a high-posterior-ascending trajectory of depressive symptoms was associated with a higher risk of CVD (2.93, 1.75–4.92; *P* for interaction = 0.027; Supplementary Table S21). No significant subgroup differences were observed for multimorbidity status, BRI, or cognitive function trajectories.

### Comparative predictive value of variable groups for CVD risk

**Figure 5:**
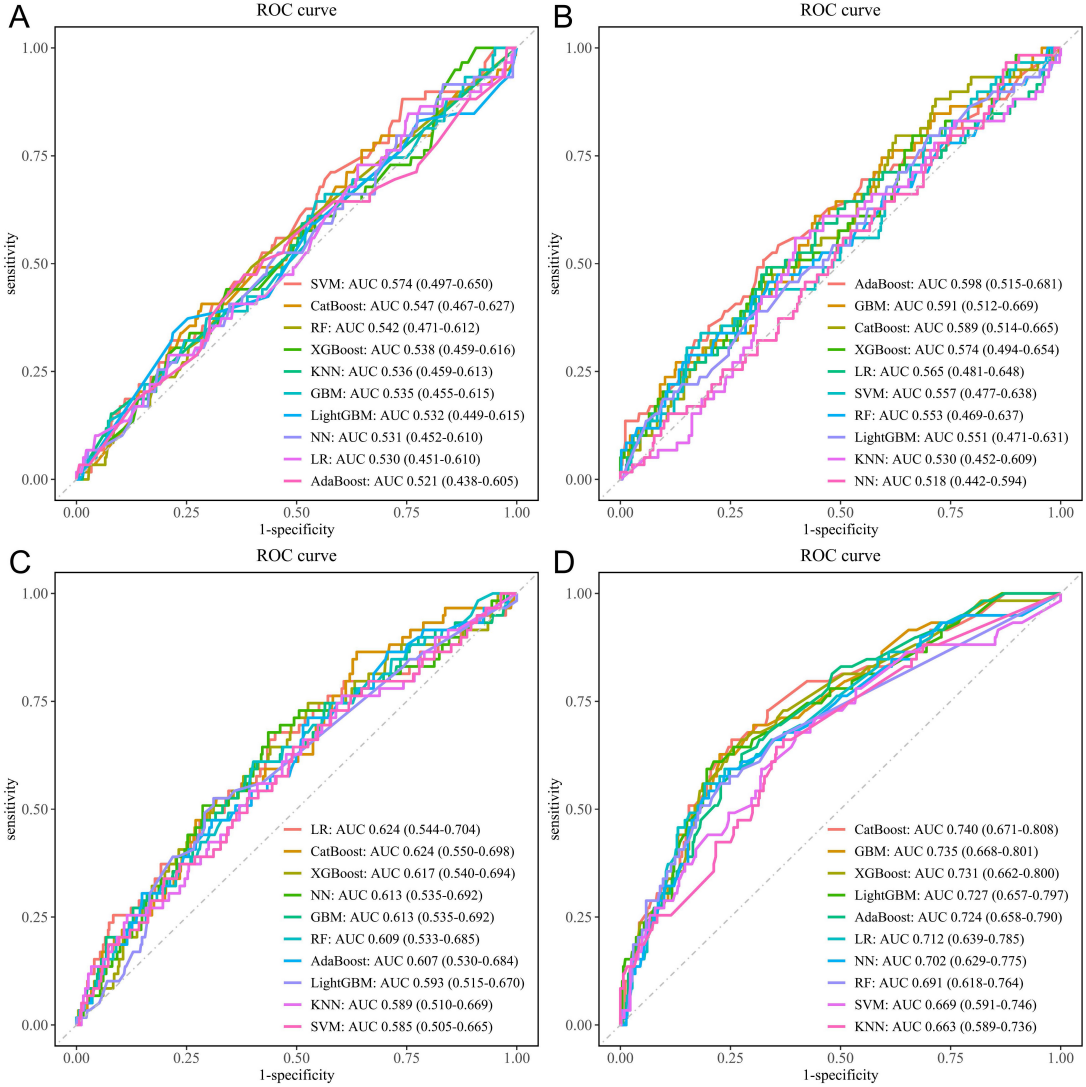
ROC curves for the ten ML models. **(A)** Sociodemographic variables only. **(B)** Blood test variables only. **(C)** Physical examination variables only. **(D)** Trajectories of health conditions variables only. ML, machine learning; LR, logistic regression; SVM, support vector machine; GBM, gradient boosting machine; NN, neural network; RF, random forest; XGBoost, extreme gradient boosting; KNN, k-nearest neighbors; AdaBoost, adaptive boosting; LightGBM, light gradient boosting machine; CatBoost, categorical boosting.

In this study, ten commonly used ML algorithms, including LR, SVM, GBM, NN, RF, XGBoost, KNN, AdaBoost, LightGBM, and CatBoost, were applied to evaluate the predictive ability of four groups of variables for CVD risk: sociodemographic variables, blood test variables, physical examination variables, and trajectories of health conditions (Supplementary Table S23). The results showed that when all variables were included, the model achieved a maximum AUC of 0.721 (95% CI: 0.655–0.787); after excluding blood test variables, model performance slightly improved (0.738, 0.678–0.798), whereas excluding trajectories of health conditions led to a marked decrease in performance (0.651, 0.574–0.728); excluding physical examination or sociodemographic variables resulted in a smaller decrease in performance (physical examination: 0.710, 0.640–0.781; sociodemographic: 0.722, 0.656–0.787). Detailed results are provided in Supplementary Table S24. Further analysis showed that the model based solely on trajectories of health conditions achieved the highest AUC among all variable groups (CatBoost: AUC 0.740, 95% CI: 0.671–0.808; Figure 5), further confirming the incremental value of trajectories of health conditions for CVD risk prediction.

### Model explanation

**Figure 6:**
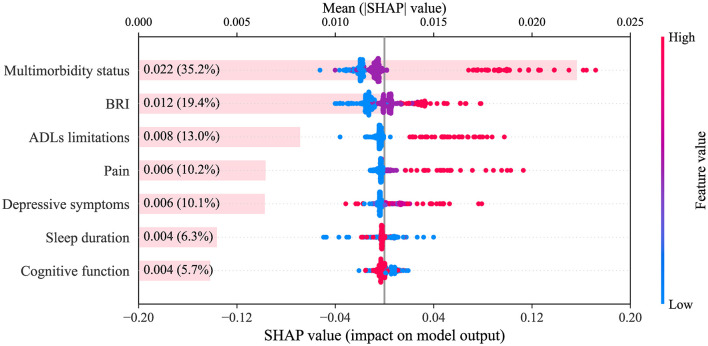
SHAP summary plot. The plot displays the relative importance and SHAP value distribution for each health condition trajectory (categorical variable) in the CatBoost model. The *x*-axis shows SHAP values. Colors correspond to distinct trajectory categories for each health condition: multimorbidity status (blue = low-ascending, purple = moderate-ascending, red = high-ascending), ADLs limitations (blue = low-stable, red = high-ascending), BRI (blue = low-stable, purple = moderate-stable, red = high-stable), pain (blue = low-stable, purple = moderate-ascending, red = high-ascending), sleep duration (blue = low-ascending, red = high-stable), depressive symptoms (blue = low-stable, light purple = moderate-descending, purple = low-ascending, red = high-posterior-ascending), cognitive function (blue = low-decreasing, red = high-stable). SHAP, SHapley Additive exPlanations; CatBoost, categorical boosting; BRI, body roundness index; ADLs, activities of daily living.

To further clarify the contribution of each trajectory of health conditions to CVD risk prediction, SHAP analysis was performed on the CatBoost model, which achieved the highest AUC in the testing set. As shown in Figure 6, multimorbidity status had the greatest impact on model output (35.2% of the total mean absolute SHAP value), followed by BRI (19.4%), ADLs limitations (13.0%), pain (10.2%), depressive symptoms (10.1%), sleep duration (6.3%), and cognitive function (5.7%). The SHAP summary plot illustrates the direction and magnitude of the impact of each trajectory category on CVD risk prediction. The high-ascending trajectory (red dots) of multimorbidity status markedly increased predicted risk, whereas the moderate-ascending trajectory (purple dots) had little effect or showed a weak protective effect, and the low-ascending trajectory (blue dots) conferred a clear protective effect. For BRI, the high-stable trajectory (red dots) was associated with increased risk, the moderate-stable trajectory (purple dots) was close to zero with a weak impact, and the low-stable trajectory (blue dots) conferred a protective effect. For ADLs limitations, the high-ascending trajectory (red dots) indicated increased risk, while the low-stable trajectory (blue dots) indicated a protective association. These findings highlight that adverse long-term trajectories of multimorbidity status, BRI, and ADLs limitations are significant contributors to CVD risk and should be incorporated into risk stratification and early preventive strategies in clinical practice.

## Discussion

In this large, prospective cohort study of middle-aged and older Chinese adults, we utilized multiple waves of CHARLS data to construct longitudinal trajectories of health conditions, including multimorbidity status, ADLs limitations, BRI, pain, sleep duration, depressive symptoms, and cognitive function. The study found that trajectories of multimorbidity status (moderate-ascending and high-ascending), ADLs limitations (high-ascending), BRI (high-stable), pain (moderate-ascending and high-ascending), depressive symptoms (moderate-descending, low-ascending, and high-posterior-ascending), and sleep duration (low-ascending) were independently associated with a significantly increased risk of CVD. In contrast, the association between declining cognitive function and CVD risk was no longer significant after adjustment for confounding factors. Incorporating these health condition trajectories into ML models significantly improved the AUC for CVD risk prediction. Further SHAP analysis revealed the importance of multimorbidity status and BRI in the model. These findings highlight the importance of dynamically monitoring health status and provide valuable insights for the precise risk stratification of CVD among middle-aged and older adults.

With advancing age, higher trajectories of multimorbidity status, ADLs limitations, and BRI are associated with an increased risk of CVD, consistent with findings from previous cohort studies (47, 48). The continuous accumulation of multimorbidity status is closely associated with the decline in functional status, and the risk of transitioning from independence to limitation in ADLs increases accordingly (49). Moreover, abdominal obesity, as indicated by a moderate or high level of BRI, has been associated with greater disease burden and functional deterioration (50), as well as a higher prevalence of CVD (51). The combined effects of these adverse trajectories of health conditions may further increase the risk of CVD. Mechanistically, these trajectories mainly result from physiological changes related to aging. As age increases, the functions of multiple organ systems and the immune system gradually decline, leading to reduced immune responses to pathogens and a tendency toward chronic low-grade inflammation, which is a key driver of the onset and progression of CVD (52, 53). At the same time, cardiac function, vascular structure, and integrity also decline with age, further increasing susceptibility to CVD (54, 55). Abdominal obesity, especially increased visceral fat, further promotes this process by enhancing the secretion of pro-inflammatory cytokines, increasing oxidative stress (56), and causing direct myocardial damage (57). Chronic inflammation, immunosenescence, and physiological deterioration form a vicious cycle, significantly amplifying the susceptibility of older adults to CVD.

Among middle-aged and older adults with moderate-ascending and high-ascending trajectories of pain, the risk of CVD is significantly increased. Previous cross-sectional and cohort studies have shown that chronic pain is associated with an increased risk of CVD (58, 59). Activation of the sympathetic nervous system is considered a potential physiological mechanism by which pain influences the development of CVD (60), and two-sample Mendelian randomization studies have also found that widespread chronic pain may be an important determinant of coronary artery disease (61). In addition, smoking has a significant impact on both pain trajectories and CVD risk. Although short-term nicotine exposure exhibits certain analgesic effects (62), smokers report pain in multiple anatomical sites more frequently than non-smokers (63, 64). Cigarette smoke can affect the regulation of various hormones and alter nociceptive pathways, thereby enhancing pain sensitivity and perception (65). Studies have also indicated a bidirectional relationship between tobacco use and persistent pain (66), and smoking is also an important contributor to CVD risk (67). These findings suggest that the interactions among pain, smoking, and CVD risk may be realized through multiple biological and behavioral pathways. Further research is needed to explore these mechanisms.

We observed that middle-aged and older adults with a low-ascending trajectory of sleep duration had a significantly increased risk of CVD. This association is consistent with findings from the UK Biobank, a large-scale prospective cohort study, which demonstrated that short sleep duration is associated with an increased risk of CVD incidence and mortality (68). Mechanistically, insufficient sleep duration may promote the development and progression of cardiovascular disease through multiple pathways, including activation of the sympathetic nervous system, exacerbation of chronic inflammation, and metabolic dysregulation (69–71). Further analysis indicated that marital status plays an important moderating role in the relationship between the trajectory of sleep duration and CVD risk. Married individuals are more likely to develop healthy sleep behaviors due to spousal support, while unmarried individuals are more prone to psychological stress, which may indirectly increase the risk of CVD (72, 73). These findings suggest that integrating the trajectory characteristics of sleep duration and relevant social behavioral factors may help optimize cardiovascular disease risk stratification and intervention strategies.

This study found that individuals with moderate-descending, low-ascending, and high-posterior-ascending trajectories of depressive symptoms had a significantly increased risk of CVD. Extensive epidemiological evidence has clearly demonstrated that higher levels of depressive symptoms are independently associated with an increased risk of cardiovascular disease (10, 74, 75). The relationship between depression and CVD is highly complex, involving mechanisms such as autonomic nervous system imbalance, chronic inflammation, unhealthy lifestyle behaviors, and various adverse metabolic factors (76–78). Further analysis suggested sex differences in the association between depressive symptoms and CVD. Previous research indicates that this association is present in both men and women, but appears stronger in women (79, 80). The stronger association between depressive symptoms and CVD observed in women may be explained by several interrelated mechanisms. Women often demonstrate greater hypothalamic–pituitary–adrenal axis and autonomic reactivity to psychosocial stress, which can amplify endothelial dysfunction and inflammatory activation in the setting of depression (81). Sex hormones, particularly estrogen, modulate vascular tone, lipid metabolism, and platelet function; their decline after menopause may exacerbate the cardiovascular impact of depression (82). In addition, sex chromosome–linked genetic and epigenetic factors may further contribute to differential susceptibility, potentially interacting with depression to accelerate CVD development (83).

After adjusting for covariates, this study found no significant association between the low-decreasing trajectory of cognitive function and CVD risk. Although some studies based on single measurements have shown an association between cognitive decline and CVD risk (11), single-point assessments are easily influenced by short-term factors such as acute health conditions and mood fluctuations, making it difficult to capture the long-term evolution of cognitive function. In addition, confounding factors such as depression and social support are often insufficiently controlled, which may lead to an overestimation of the association. The results of this study suggest that this discrepancy may also be related to differences in analytical methods, follow-up duration, and sample characteristics. Furthermore, longitudinal neuroimaging evidence indicates that vascular injury, metabolic disturbances, and systemic inflammation are shared biological pathways linking cognitive decline and CVD, and that variation in these mechanisms across populations and study designs may partly account for inconsistent findings (84). Future studies should rely on multicenter, long-term cohort data to further clarify the potential association between changes in cognitive function and CVD risk, in order to facilitate more accurate identification and intervention of cardiovascular risk.

Given that CVD often has a prolonged preclinical phase, there is an urgent need for effective tools to enable early identification of high-risk individuals. In this study, we developed a CVD risk prediction model that integrates trajectories of seven physiological and psychological health conditions. Compared to traditional models that include only sociodemographic, blood test, and physical examination variables, the inclusion of health condition trajectories significantly improved the model's predictive ability. By dynamically integrating multidimensional health data, this model can more comprehensively capture long-term changes in both physiological and psychological health, providing a new perspective for the early identification and intervention of CVD risk. Compared with models that rely on coronary angiography, cardiac magnetic resonance imaging, or multi-omics technologies (85–87), our model demonstrates greater accessibility and broader application prospects. All relevant information can be conveniently obtained through standardized questionnaires, physical examinations, and routine blood tests, making it particularly suitable for screening high-risk individuals for CVD in community populations. In addition, this approach relies on low-cost and easily accessible data, which is especially valuable for implementation in resource-limited settings.

The major strength of this study lies in its ability to identify distinct trajectories of multimorbidity status, ADLs limitations, BRI, pain, sleep duration, depressive symptoms, and cognitive function, and to systematically elucidate their associations with the risk of incident CVD as well as their predictive value. However, this study also has several limitations. First, the study population was limited to Chinese individuals, which restricts the external generalizability of the findings. Second, the assessments of CVD and multimorbidity status were based on self-reported physician-diagnosed conditions, without standardized diagnostic verification, which may introduce recall or misclassification bias. In addition, ADLs limitations, pain, sleep duration, depressive symptoms, and cognitive function were all measured using self-reported methods, which, although commonly employed in large-scale epidemiological studies, may still lead to subjective bias. Third, while the SHAP method improved model interpretability, its analytical results only reflect associations between variables and cannot be used for causal inference. Finally, there may still be unmeasured or residual confounding factors. Future research should validate these findings in multi-center and multi-ethnic populations to enhance generalizability, incorporate more objective assessment tools to reduce subjective bias, and link survey data with clinical or health insurance records to enable outcome validation, thereby strengthening the robustness and applicability of the conclusions.

## Conclusion

This study demonstrates that the long-term deterioration of health conditions, including multimorbidity status, ADLs limitations, BRI, pain, sleep duration, and depressive symptoms, is associated with an increased risk of CVD in middle-aged and older adults. The ML model based on trajectories of health condition significantly improves the accuracy of CVD risk prediction, providing an efficient and cost-effective tool for early screening and intervention, with significant clinical applicability.

## Data Availability

Publicly available datasets were analyzed in this study. This data can be found here: http://charls.pku.edu.cn.
