# Supplementary material for: Trajectories of health conditions predict cardiovascular disease risk among middle-aged and older adults: a national cohort study
Source: Front Nutr. 2025 Sep 12;12:1657587. doi: 10.3389/fnut.2025.1657587 (PMC12463975; doi:10.3389/fnut.2025.1657587)
Supplement: Supplementary file 1 [file Table_1.docx]

**Supplementary Material**

**Table S1.** Laboratory assay methods, quality control, and detection limits in CHARLS

**Table S2.** The classification fitting information of each latent class by the LCGM (multimorbidity status)

**Table S3.** The classification fitting information of each latent class by the LCGM (ADLs limitations)

**Table S4.** The classification fitting information of each latent class by the LCGM (BRI)

**Table S5.** The classification fitting information of each latent class by the LCGM (pain)

**Table S6.** The classification fitting information of each latent class by the LCGM (sleep duration)

**Table S7.** The classification fitting information of each latent class by the LCGM (depressive symptoms)

**Table S8.** The classification fitting information of each latent class by the LCGM (cognitive function)

**Table S9.** Characteristics of participants according to multimorbidity status trajectories

**Table S10.** Characteristics of participants according to ADLs limitations trajectories

**Table S11.** Characteristics of participants according to BRI trajectories

**Table S12.** Characteristics of participants according to pain trajectories

**Table S13.** Characteristics of participants according to sleep duration trajectories

**Table S14.** Characteristics of participants according to depressive symptoms trajectories

**Table S15.** Characteristics of participants according to cognitive function trajectories

**Table S16.** Subgroup analysis of the relationship between multimorbidity status trajectories and CVD risk

**Table S17.** Subgroup analysis of the relationship between ADLs limitations trajectories and CVD risk

**Table S18.** Subgroup analysis of the relationship between BRI trajectories and CVD risk

**Table S19.** Subgroup analysis of the relationship between pain trajectories and CVD risk

**Table S20.** Subgroup analysis of the relationship between sleep duration trajectories and CVD risk

**Table S21.** Subgroup analysis of the relationship between depressive symptoms trajectories and CVD risk

**Table S22.** Subgroup analysis of the relationship between cognitive function trajectories and CVD risk

**Table S23.** Classification of variables

**Table S24.** AUC of ten ML algorithms across different variable groups

**Fig.S1.** Trajectories of cognitive function

**Table S1.** Laboratory assay methods, quality control, and detection limits in CHARLS

| Biomarker | Assay Method | CV%  (Within-assay) | CV%  (Between-assay) | Detection Limit |
| --- | --- | --- | --- | --- |
| HbA1C (%) | High performance liquid chromatography | <1.16 | <1.27 | 3.85–17.80 |
| hsCRP (mg/L) | Immunoturbidimetric assay | <2.94 | <2.09 | 0.10–140.00 |
| TC (mmol/L) | Oxidase method | <0.70 | <3.18 | 0.13–18.18 |
| HDL-C (mmol/L) | Direct method | <0.67 | <3.14 | 0.09–3.08 |
| LDL-C (mmol/L) | Direct method | <1.38 | <2.90 | 0.22–14.14 |
| TG (mmol/L) | Oxidase method | <0.41 | <3.64 | 0.12–9.78 |
| GLU (mmol/L) | Hexokinase | <0.40 | <2.50 | 0.13–39.58 |
| UA (μmol/L) | Uricase, catalase | <0.25 | <2.32 | 38.50–1044.00 |
| CysC (mg/L) | Immunoturbidimetric assay | <1.84 | <1.87 | 0.10–6.00 |
| BUN (mmol/L) | Enzymatic | <0.71 | <2.82 | 0.65–39.05 |
| CREA (μmol/L) | Picric acid method | <1.87 | <4.34 | 22.85–2151.70 |

HbA1C, hemoglobin A1c; hsCRP, high-sensitivity C-reactive protein; TC, total cholesterol; HDL-C, high-density lipoprotein cholesterol; LDL-C, low-density lipoprotein cholesterol; TG, triglycerides; GLU, fasting blood glucose; UA, uric acid; CysC, cystatin C; BUN, blood urea nitrogen; CREA, creatinine.

**Table S2.** The classification fitting information of each latent class by the LCGM (multimorbidity status)

| **Estimation method** | **AIC** | **BIC** | **aBIC** | **Entropy** | **LMR(*P*)** | **BLRT(*P*)** | **Class probability** |
| --- | --- | --- | --- | --- | --- | --- | --- |
| Linear estimation |  |  |  |  |  |  |  |
| CLASS-1 | 23629.288 | 23658.433 | 23642.546 | / | / | / | 1.000 |
| CLASS-2 | 19217.210 | 19263.840 | 19238.422 | 0.944 | <0.001 | <0.001 | 0.708/0.292 |
| CLASS-3 | 17363.621 | 17427.738 | 17392.788 | 0.939 | <0.001 | <0.001 | 0.116/0.491/0.393 |
| CLASS-4 | 14428.566 | 14510.169 | 14465.688 | 0.999 | 0.323 | <0.001 | 0.087/0.181/0.403/0.329 |
| CLASS-5 | 10302.337 | 10401.428 | 10347.414 | 1.000 | 0.648 | <0.001 | 0.024/0.403/0.329/0.063/0.181 |
| Quadratic estimation |  |  |  |  |  |  |  |
| CLASS-1 | 20273.806 | 20314.608 | 20292.367 | / | / | / | 1.000 |
| CLASS-2 | 17637.210 | 17701.328 | 17666.378 | 0.941 | <0.001 | <0.001 | 0.276/0.724 |
| **CLASS-3** | **16380.419** | **16467.851** | **16420.193** | **0.940** | **0.029** | **<0.001** | **0.496/0.110/0.395** |
| CLASS-4 | 13478.348 | 13589.096 | 13528.728 | 1.000 | 0.237 | <0.001 | 0.329/0.087/0.181/0.403 |
| CLASS-5 | 13382.001 | 13516.064 | 13442.987 | 0.993 | 0.496 | <0.001 | 0.403/0.087/0.329/0.015/0.166 |
| Free estimation |  |  |  |  |  |  |  |
| CLASS-1 | 23611.474 | 23646.447 | 23627.384 | / | / | / | 1.000 |
| CLASS-2 | 19170.911 | 19223.370 | 19194.775 | 0.944 | <0.001 | <0.001 | 0.708/0.292 |
| CLASS-3 | 17291.210 | 17361.156 | 17323.029 | 0.940 | <0.001 | <0.001 | 0.393/0.491/0.116 |
| CLASS-4 | 14329.632 | 14417.065 | 14369.406 | 0.999 | 0.336 | <0.001 | 0.182/0.403/0.087/0.329 |
| CLASS-5 | 10944.827 | 11049.746 | 10992.556 | 1.000 | 0.373 | <0.001 | 0.038/0.328/0.202/0.351/0.082 |

**Note:** The **bolded** row represents the optimal trajectory model. The selection was based on the following criteria: lowest AIC, BIC, and aBIC values; statistically significant P-values for BLRT and LMR tests (P < 0.05); high entropy (≥ 0.8); and each class comprising at least 5% of the total sample. Additionally, the distinctiveness and interpretability of the trajectories were carefully considered.

**Table S3.** The classification fitting information of each latent class by the LCGM (ADLs limitations)

| **Estimation method** | **AIC** | **BIC** | **aBIC** | **Entropy** | **LMR(*P*)** | **BLRT(*P*)** | **Class probability** |
| --- | --- | --- | --- | --- | --- | --- | --- |
| Linear estimation |  |  |  |  |  |  |  |
| CLASS-1 | 22336.132 | 22365.276 | 22349.390 | / | / | / | 1.000 |
| CLASS-2 | 18436.333 | 18482.963 | 18457.545 | 0.990 | <0.001 | <0.001 | 0.060/0.940 |
| CLASS-3 | 17227.004 | 17291.121 | 17256.171 | 0.986 | 0.262 | <0.001 | 0.019/0.077/0.904 |
| CLASS-4 | 16492.566 | 16574.170 | 16529.688 | 0.989 | 0.012 | <0.001 | 0.029/0.881/0.079/0.011 |
| CLASS-5 | 15746.588 | 15845.678 | 15791.665 | 0.989 | 0.368 | <0.001 | 0.009/0.876/0.066/0.021/0.029 |
| Quadratic estimation |  |  |  |  |  |  |  |
| CLASS-1 | 20688.779 | 20729.581 | 20707.340 | / | / | / | 1.000 |
| CLASS-2 | 18231.254 | 18295.371 | 18260.421 | 0.987 | 0.061 | <0.001 | 0.058/0.942 |
| CLASS-3 | 17085.527 | 17172.960 | 17125.301 | 0.991 | 0.020 | <0.001 | 0.079/0.025/0.025 |
| CLASS-4 | 16109.579 | 16220.327 | 16159.959 | 0.993 | 0.497 | <0.001 | 0.039/0.012/0.894/0.056 |
| CLASS-5 | 15449.149 | 15583.212 | 15510.135 | 0.994 | 0.196 | <0.001 | 0.040/0.004/0.050/0.016/0.890 |
| Free estimation |  |  |  |  |  |  |  |
| CLASS-1 | 22337.915 | 22372.888 | 22353.824 | / | / | / | 1.000 |
| **CLASS-2** | **18391.515** | **18443.975** | **18415.379** | **0.988** | **<0.001** | **<0.001** | **0.068/0.932** |
| CLASS-3 | 17118.209 | 17188.155 | 17150.028 | 0.988 | 0.072 | <0.001 | 0.022/0.895/0.083 |
| CLASS-4 | 16342.598 | 16430.031 | 16382.372 | 0.992 | 0.602 | <0.001 | 0.050/0.051/0.886/0.013 |
| CLASS-5 | 15618.219 | 15723.138 | 15665.947 | 0.993 | 0.663 | <0.001 | 0.007/0.014/0.052/0.049/0.878 |

**Note:** The **bolded** row represents the optimal trajectory model. The selection was based on the following criteria: lowest AIC, BIC, and aBIC values; statistically significant P-values for BLRT and LMR tests (P < 0.05); high entropy (≥ 0.8); and each class comprising at least 5% of the total sample. Additionally, the distinctiveness and interpretability of the trajectories were carefully considered.

**Table S4.** The classification fitting information of each latent class by the LCGM (BRI)

| **Estimation method** | **AIC** | **BIC** | **aBIC** | **Entropy** | **LMR(P)** | **BLRT(P)** | **Class probability** |
| --- | --- | --- | --- | --- | --- | --- | --- |
| Linear estimation |  |  |  |  |  |  |  |
| CLASS-1 | 25354.400 | 25383.544 | 25367.658 | / | / | / | 1.000 |
| CLASS-2 | 21961.095 | 22007.726 | 21982.308 | 0.834 | <0.001 | <0.001 | 0.379/0.621 |
| CLASS-3 | 20513.086 | 20577.203 | 20542.254 | 0.849 | <0.001 | <0.001 | 0.140/0.415/0.445 |
| CLASS-4 | 19938.837 | 20020.440 | 19975.959 | 0.811 | <0.001 | <0.001 | 0.371/0.279/0.096/0.254 |
| CLASS-5 | 19718.527 | 19817.617 | 19763.604 | 0.788 | 0.013 | <0.001 | 0.273/0.325/0.057/0.149/0.196 |
| Quadratic estimation |  |  |  |  |  |  |  |
| CLASS-1 | 22850.591 | 22891.393 | 22869.152 | / | / | / | 1.000 |
| CLASS-2 | 20998.849 | 21062.966 | 21028.016 | 0.795 | <0.001 | <0.001 | 0.657/0.343 |
| CLASS-3 | 20109.207 | 20196.640 | 20148.981 | 0.823 | <0.001 | <0.001 | 0.125/0.415/0.461 |
| CLASS-4 | 19723.683 | 19834.430 | 19774.062 | 0.789 | <0.001 | <0.001 | 0.090/0.383/0.266/0.261 |
| CLASS-5 | 19493.162 | 19627.226 | 19554.149 | 0.815 | 0.002 | <0.001 | 0.092/0.267/0.372/0.254/0.015 |
| Free estimation |  |  |  |  |  |  |  |
| CLASS-1 | 25329.257 | 25364.230 | 25345.167 | / | / | / | 1.000 |
| CLASS-2 | 21903.839 | 21956.298 | 21927.703 | 0.835 | <0.001 | <0.001 | 0.620/0.380 |
| **CLASS-3** | **20429.699** | **20499.645** | **20461.518** | **0.851** | **<0.001** | **<0.001** | **0.140/0.412/0.448** |
| CLASS-4 | 19841.642 | 19929.074 | 19881.416 | 0.813 | <0.001 | <0.001 | 0.368/0.252/0.098/0.282 |
| CLASS-5 | 19609.381 | 19714.300 | 19657.109 | 0.791 | 0.008 | <0.001 | 0.058/0.151/0.326/0.191/0.273 |

**Note:** The **bolded** row represents the optimal trajectory model. The selection was based on the following criteria: lowest AIC, BIC, and aBIC values; statistically significant P-values for BLRT and LMR tests (P < 0.05); high entropy (≥ 0.8); and each class comprising at least 5% of the total sample. Additionally, the distinctiveness and interpretability of the trajectories were carefully considered.

**Table S5.** The classification fitting information of each latent class by the LCGM (pain)

| **Estimation method** | **AIC** | **BIC** | **aBIC** | **Entropy** | **LMR(*P*)** | **BLRT(*P*)** | **Class probability** |
| --- | --- | --- | --- | --- | --- | --- | --- |
| Linear estimation |  |  |  |  |  |  |  |
| CLASS-1 | 32963.225 | 32992.369 | 32976.483 | / | / | / | 1.000 |
| CLASS-2 | 30696.773 | 30743.404 | 30717.986 | 0.981 | <0.001 | <0.001 | 0.904/0.096 |
| CLASS-3 | 29540.098 | 29604.215 | 29569.266 | 0.985 | <0.001 | <0.001 | 0.101/0.843/0.056 |
| CLASS-4 | 28619.922 | 28701.526 | 28657.044 | 0.983 | 0.002 | <0.001 | 0.055/0.032/0.834/0.080 |
| CLASS-5 | 28164.364 | 28263.454 | 28209.440 | 0.980 | 0.003 | <0.001 | 0.035/0.072/0.054/0.806/0.032 |
| Quadratic estimation |  |  |  |  |  |  |  |
| CLASS-1 | 32579.810 | 32620.612 | 32598.371 | / | / | / | 1.000 |
| CLASS-2 | 29973.108 | 30037.225 | 30002.275 | 0.983 | <0.001 | <0.001 | 0.099/0.901 |
| CLASS-3 | 28528.175 | 28615.607 | 28567.948 | 0.987 | <0.001 | <0.001 | 0.083/0.030/0.887 |
| CLASS-4 | 27679.521 | 27790.269 | 27729.901 | 0.982 | 0.026 | <0.001 | 0.847/0.028/0.082/0.043 |
| CLASS-5 | 26852.060 | 26986.123 | 26913.046 | 0.982 | 0.204 | <0.001 | 0.079/0.041/0.840/0.031/0.009 |
| Free estimation |  |  |  |  |  |  |  |
| CLASS-1 | 32891.185 | 32926.159 | 32907.095 | / | / | / | 1.000 |
| CLASS-2 | 30027.100 | 30079.560 | 30050.964 | 0.982 | <0.001 | <0.001 | 0.901/0.099 |
| **CLASS-3** | **28632.037** | **28701.983** | **28663.856** | **0.988** | **<0.001** | **<0.001** | **0.101/0.843/0.056** |
| CLASS-4 | 27928.931 | 28016.364 | 27968.705 | 0.983 | 0.300 | <0.001 | 0.854/0.022/0.080/0.044 |
| CLASS-5 | 27079.337 | 27184.256 | 27127.065 | 0.981 | 0.242 | <0.001 | 0.041/0.010/0.836/0.079/0.034 |

**Note:** The **bolded** row represents the optimal trajectory model. The selection was based on the following criteria: lowest AIC, BIC, and aBIC values; statistically significant P-values for BLRT and LMR tests (P < 0.05); high entropy (≥ 0.8); and each class comprising at least 5% of the total sample. Additionally, the distinctiveness and interpretability of the trajectories were carefully considered.

**Table S6.** The classification fitting information of each latent class by the LCGM (sleep duration)

| **Estimation method** | **AIC** | **BIC** | **aBIC** | **Entropy** | **LMR(*P*)** | **BLRT(*P*)** | **Class probability** |
| --- | --- | --- | --- | --- | --- | --- | --- |
| Linear estimation |  |  |  |  |  |  |  |
| CLASS-1 | 29416.802 | 29445.946 | 29430.060 | / | / | / | 1.000 |
| CLASS-2 | 28407.627 | 28454.258 | 28428.840 | 0.680 | <0.001 | <0.001 | 0.771/0.229 |
| CLASS-3 | 28107.171 | 28171.288 | 28136.338 | 0.646 | <0.001 | <0.001 | 0.085/0.372/0.543 |
| CLASS-4 | 28071.895 | 28153.499 | 28109.017 | 0.689 | <0.001 | <0.001 | 0.080/0.009/0.431/0.480 |
| CLASS-5 | 28044.526 | 28143.616 | 28089.603 | 0.724 | 0.008 | <0.001 | 0.014/0.008/0.469/0.080/0.429 |
| Quadratic estimation |  |  |  |  |  |  |  |
| CLASS-1 | 28807.312 | 28848.114 | 28825.874 | / | / | / | 1.000 |
| **CLASS-2** | **28197.205** | **28261.322** | **28226.372** | **0.698** | **<0.001** | **<0.001** | **0.801/0.199** |
| CLASS-3 | 28060.550 | 28147.983 | 28100.324 | 0.580 | 0.011 | <0.001 | 0.082/0.510/0.407 |
| CLASS-4 | 27996.226 | 28106.974 | 28046.606 | 0.761 | 0.011 | <0.001 | 0.368/0.517/0.036/0.080 |
| CLASS-5 | 27915.191 | 28049.254 | 27976.177 | 0.799 | <0.001 | <0.001 | 0.286/0.049/0.038/0.486/0.141 |
| Free estimation |  |  |  |  |  |  |  |
| CLASS-1 | 29399.568 | 29434.541 | 29415.477 | / | / | / | 1.000 |
| CLASS-2 | 28379.203 | 28431.663 | 28403.067 | 0.684 | <0.001 | <0.001 | 0.228/0.772 |
| CLASS-3 | 28076.901 | 28146.847 | 28108.720 | 0.648 | <0.001 | <0.001 | 0.374/0.084/0.542 |
| CLASS-4 | 28020.407 | 28107.840 | 28060.181 | 0.724 | <0.001 | <0.001 | 0.084/0.008/0.374/0.534 |
| CLASS-5 | 27978.834 | 28083.753 | 28026.562 | 0.727 | 0.031 | <0.001 | 0.436/0.009/0.079/0.458/0.018 |

**Note:** The **bolded** row represents the optimal trajectory model. The selection was based on the following criteria: lowest AIC, BIC, and aBIC values; statistically significant P-values for BLRT and LMR tests (P < 0.05); an acceptable entropy value (0.698); and each class comprising at least 5% of the total sample. Additionally, the distinctiveness and interpretability of the trajectories were carefully considered.

**Table S7.** The classification fitting information of each latent class by the LCGM (depressive symptoms)

| **Estimation method** | **AIC** | **BIC** | **aBIC** | **Entropy** | **LMR(*P*)** | **BLRT(*P*)** | **Class probability** |
| --- | --- | --- | --- | --- | --- | --- | --- |
| Linear estimation |  |  |  |  |  |  |  |
| CLASS-1 | 47254.525 | 47283.669 | 47267.782 | / | / | / | 1.000 |
| CLASS-2 | 45581.012 | 45627.642 | 45602.224 | 0.836 | <0.001 | <0.001 | 0.187/0.813 |
| CLASS-3 | 45261.502 | 45325.619 | 45290.669 | 0.761 | <0.001 | <0.001 | 0.064/0.277/0.658 |
| CLASS-4 | 45035.212 | 45116.815 | 45072.334 | 0.799 | <0.001 | <0.001 | 0.063/0.127/0.662/0.148 |
| CLASS-5 | 44964.136 | 45063.226 | 45009.213 | 0.806 | 0.393 | <0.001 | 0.074/0.128/0.013/0.140/0.645 |
| Quadratic estimation |  |  |  |  |  |  |  |
| CLASS-1 | 46470.263 | 46511.065 | 46488.824 | / | / | / | 1.000 |
| CLASS-2 | 45380.674 | 45444.791 | 45409.842 | 0.840 | <0.001 | <0.001 | 0.168/0.832 |
| CLASS-3 | 45161.927 | 45249.360 | 45201.701 | 0.842 | 0.002 | <0.001 | 0.787/0.076/0.137 |
| **CLASS-4** | **44872.975** | **44983.723** | **44923.355** | **0.825** | **<0.001** | **<0.001** | **0.053/0.117/0.135/0.694** |
| CLASS-5 | 44700.312 | 44834.375 | 44761.298 | 0.836 | 0.150 | <0.001 | 0.104/0.042/0.050/0.101/0.703 |
| Free estimation |  |  |  |  |  |  |  |
| CLASS-1 | 47254.906 | 47289.879 | 47270.815 | / | / | / | 1.000 |
| CLASS-2 | 45576.397 | 45628.856 | 45600.261 | 0.835 | <0.001 | <0.001 | 0.191/0.809 |
| CLASS-3 | 45229.184 | 45299.130 | 45261.003 | 0.773 | <0.001 | <0.001 | 0.062/0.666/0.272 |
| CLASS-4 | 45008.198 | 45095.631 | 45047.972 | 0.800 | 0.015 | <0.001 | 0.669/0.155/0.057/0.119 |
| CLASS-5 | 44879.066 | 44983.985 | 44926.795 | 0.763 | 0.011 | <0.001 | 0.273/0.037/0.545/0.043/0.102 |

**Note:** The **bolded** row represents the optimal trajectory model. The selection was based on the following criteria: lowest AIC, BIC, and aBIC values; statistically significant P-values for BLRT and LMR tests (P < 0.05); high entropy (≥ 0.8); and each class comprising at least 5% of the total sample. Additionally, the distinctiveness and interpretability of the trajectories were carefully considered.

**Table S8.** The classification fitting information of each latent class by the LCGM (cognitive function)

| **Estimation method** | **AIC** | **BIC** | **aBIC** | **Entropy** | **LMR(*P*)** | **BLRT(*P*)** | **Class probability** |
| --- | --- | --- | --- | --- | --- | --- | --- |
| Linear estimation |  |  |  |  |  |  |  |
| CLASS-1 | 43195.700 | 43224.844 | 43208.958 | / | / | / | 1.000 |
| CLASS-2 | 41720.732 | 41767.363 | 41741.945 | 0.710 | <0.001 | <0.001 | 0.341/0.659 |
| CLASS-3 | 41360.448 | 41424.566 | 41389.616 | 0.670 | <0.001 | <0.001 | 0.556/0.262/0.182 |
| CLASS-4 | 41294.821 | 41376.425 | 41331.943 | 0.691 | <0.001 | <0.001 | 0.533/0.240/0.200/0.026 |
| CLASS-5 | 41284.517 | 41383.607 | 41329.593 | 0.724 | 0.018 | <0.001 | 0.200/0.006/0.028/0.237/0.529 |
| Quadratic estimation |  |  |  |  |  |  |  |
| CLASS-1 | 42230.008 | 42270.809 | 42248.569 | / | / | / | 1.000 |
| CLASS-2 | 41491.534 | 41555.651 | 41520.702 | 0.626 | <0.001 | <0.001 | 0.346/0.654 |
| CLASS-3 | 41300.467 | 41387.899 | 41340.240 | 0.615 | <0.001 | <0.001 | 0.270/0.561/0.169 |
| CLASS-4 | 41271.165 | 41381.913 | 41321.545 | 0.672 | 0.002 | <0.001 | 0.229/0.022/0.544/0.205 |
| CLASS-5 | 41259.846 | 41393.909 | 41320.832 | 0.706 | 0.009 | 0.005 | 0.209/0.005/0.541/0.222/0.023 |
| Free estimation |  |  |  |  |  |  |  |
| CLASS-1 | 43186.228 | 43221.201 | 43202.137 | / | / | / | 1.000 |
| **CLASS-2** | **41711.236** | **41763.695** | **41735.100** | **0.708** | **<0.001** | **<0.001** | **0.656/0.344** |
| CLASS-3 | 41351.895 | 41421.841 | 41383.713 | 0.670 | <0.001 | <0.001 | 0.266/0.551/0.182 |
| CLASS-4 | 41288.080 | 41375.513 | 41327.854 | 0.690 | <0.001 | <0.001 | 0.025/0.533/0.241/0.200 |
| CLASS-5 | 41276.862 | 41381.781 | 41324.590 | 0.726 | 0.022 | 0.005 | 0.530/0.239/0.029/0.198/0.004 |

**Note:** The **bolded** row represents the optimal trajectory model. The selection was based on the following criteria: lowest AIC, BIC, and aBIC values; statistically significant P-values for BLRT and LMR tests (P < 0.05); a moderate entropy value (0.708);; and each class comprising at least 5% of the total sample. Additionally, the distinctiveness and interpretability of the trajectories were carefully considered.

**Table S9.** Characteristics of participants according to multimorbidity status trajectories

| **Groups** | **Overall** | **Low-ascending** | **Moderate-ascending** | **High-ascending** | ***P*** |
| --- | --- | --- | --- | --- | --- |
| Multimorbidity status | 2512 | 991(39.45) | 1245(49.56) | 276(10.99) |  |
| Age |  |  |  |  | <0.001 |
| 45~59 | 1157 (46.06) | 531 (53.58) | 541 (43.45) | 85 (30.80) |  |
| 60~74 | 1228 (48.89) | 412 (41.57) | 640 (51.41) | 176 (63.77) |  |
| ≥75 | 127 (5.06) | 48 (4.84) | 64 (5.14) | 15 (5.43) |  |
| Gender |  |  |  |  | 0.207 |
| Female | 1140 (45.38) | 441 (44.50) | 560 (44.98) | 1140 (45.38) |  |
| Male | 1372 (54.62) | 550 (55.50) | 685 (55.02) | 1372 (54.62) |  |
| Marital status |  |  |  |  | 0.600 |
| Married | 2117 (84.28) | 832 (83.96) | 1057 (84.90) | 2117 (84.28) |  |
| Unmarried | 395 (15.72) | 159 (16.04) | 188 (15.10) | 395 (15.72) |  |
| Residence |  |  |  |  | 0.792 |
| Rural | 1649 (65.64) | 643 (64.88) | 825 (66.27) | 181 (65.58) |  |
| Urban | 863 (34.36) | 348 (35.12) | 420 (33.73) | 95 (34.42) |  |
| Education level |  |  |  |  | 0.008 |
| Primary school or lower | 1491 (59.36) | 548 (55.30) | 760 (61.04) | 183 (66.30) |  |
| Middle school | 685 (27.27) | 297 (29.97) | 327 (26.27) | 61 (22.10) |  |
| High school or above | 336 (13.38) | 146 (14.73) | 158 (12.69) | 32 (11.59) |  |
| Drinking status |  |  |  |  | 0.014 |
| Never drinking | 1543 (61.43) | 584 (58.93) | 769 (61.77) | 190 (68.84) |  |
| Drinking ≤1/week | 448 (17.83) | 200 (20.18) | 215 (17.27) | 33 (11.96) |  |
| Drinking >1/week | 521 (20.74) | 207 (20.89) | 261 (20.96) | 53 (19.20) |  |
| Smoking status |  |  |  |  | 0.066 |
| Never smoking | 1299 (51.71) | 516 (52.07) | 634 (50.92) | 149 (53.99) |  |
| Former smoking | 427 (17.00) | 145 (14.63) | 230 (18.47) | 52 (18.84) |  |
| Current smoking | 786 (31.29) | 330 (33.30) | 381 (30.60) | 75 (27.17) |  |
| BMI |  |  |  |  | 0.002 |
| <23.9 | 1391 (55.37) | 571 (57.62) | 688 (55.26) | 132 (47.83) |  |
| 24-27.9 | 809 (32.21) | 323 (32.59) | 391 (31.41) | 95 (34.42) |  |
| ≥28 | 312 (12.42) | 97 (9.79) | 166 (13.33) | 49 (17.75) |  |
| ADLs limitations |  |  |  |  | <0.001 |
| Low-stable | 2342 (93.23) | 960 (96.87) | 1149 (92.29) | 233 (84.42) |  |
| High-ascending | 170 (6.77) | 31 (3.13) | 96 (7.71) | 43 (15.58) |  |
| BRI |  |  |  |  | <0.001 |
| Low-stable | 1126 (44.82) | 474 (47.83) | 561 (45.06) | 91 (32.97) |  |
| Moderate-stable | 1035 (41.20) | 405 (40.87) | 509 (40.88) | 121 (43.84) |  |
| High-stable | 351 (13.97) | 112 (11.30) | 175 (14.06) | 64 (23.19) |  |
| Pain |  |  |  |  | <0.001 |
| Low-stable | 2118 (84.32) | 916 (92.43) | 1020 (81.93) | 182 (65.94) |  |
| Moderate-ascending | 253 (10.07) | 53 (5.35) | 141 (11.33) | 59 (21.38) |  |
| High-ascending | 141 (5.61) | 22 (2.22) | 84 (6.75) | 35 (12.68) |  |
| Sleep duration |  |  |  |  | <0.001 |
| High-stable | 2012 (80.10) | 847 (85.47) | 979 (78.63) | 186 (67.39) |  |
| Low-ascending | 500 (19.90) | 144 (14.53) | 266 (21.37) | 90 (32.61) |  |
| Depressive symptoms |  |  |  |  | <0.001 |
| Low-stable | 1744(69.43) | 776 (78.30) | 826 (66.35) | 142 (51.45) |  |
| Moderate-descending | 340(13.54) | 95 (9.59) | 192 (15.42) | 53 (19.20) |  |
| Low-ascending | 294 (11.70) | 94 (9.49) | 148 (11.89) | 52 (18.84) |  |
| High-posterior-ascending | 134 (5.33) | 26 (2.62) | 79 (6.35) | 29 (10.51) |  |
| Cognitive function |  |  |  |  | 0.051 |
| High-stable | 1648 (65.61) | 670 (67.61) | 813 (65.30) | 165 (59.78) |  |
| Low-descending | 864 (34.39) | 321 (32.39) | 432 (34.70) | 111 (40.22) |  |

BMI, body mass index; ADLs, activities of daily living; BRI, body roundness index.

**Table S10.** Characteristics of participants according to ADLs limitations trajectories

| **Groups** | **Overall** | **Low-stable** | **High-ascending** | ***P*** |
| --- | --- | --- | --- | --- |
| ADLs limitations | 2512 | 2342 | 170 |  |
| Age |  |  |  | <0.001 |
| 45~59 | 1157 (46.06) | 1115 (47.61) | 42 (24.71) |  |
| 60~74 | 1228 (48.89) | 1118 (47.74) | 110 (64.71) |  |
| ≥75 | 127 (5.06) | 109 (4.65) | 18 (10.59) |  |
| Gender |  |  |  | <0.001 |
| Female | 1140 (45.38) | 1040 (44.41) | 100 (58.82) |  |
| Male | 1372 (54.62) | 1302 (55.59) | 70 (41.18) |  |
| Marital status |  |  |  | 0.953 |
| Married | 2117 (84.28) | 1974 (84.29) | 143 (84.12) |  |
| Unmarried | 395 (15.72) | 368 (15.71) | 27 (15.88) |  |
| Residence |  |  |  | <0.001 |
| Rural | 1649 (65.64) | 1517 (64.77) | 132 (77.65) |  |
| Urban | 863 (34.36) | 825 (35.23) | 38 (22.35) |  |
| Education level |  |  |  | <0.001 |
| Primary school or lower | 1491 (59.36) | 1356 (57.90) | 135 (79.41) |  |
| Middle school | 685 (27.27) | 656 (28.01) | 29 (17.06) |  |
| High school or above | 336 (13.38) | 330 (14.09) | 6 (3.53) |  |
| Drinking status |  |  |  | <0.001 |
| Never drinking | 1543 (61.43) | 1413 (60.33) | 130 (76.47) |  |
| Drinking ≤1/week | 448 (17.83) | 430 (18.36) | 18 (10.59) |  |
| Drinking >1/week | 521 (20.74) | 499 (21.31) | 22 (12.94) |  |
| Smoking status |  |  |  | 0.077 |
| Never smoking | 1299 (51.71) | 1198 (51.15) | 101 (59.41) |  |
| Former smoking | 427 (17.00) | 399 (17.04) | 28 (16.47) |  |
| Current smoking | 786 (31.29) | 745 (31.81) | 41 (24.12) |  |
| BMI |  |  |  | 0.270 |
| <23.9 | 1391 (55.37) | 1287 (54.95) | 104 (61.18) |  |
| 24-27.9 | 809 (32.21) | 760 (32.45) | 49 (28.82) |  |
| ≥28 | 312 (12.42) | 295 (12.60) | 17 (10.00) |  |
| Multimorbidity status |  |  |  | <0.001 |
| Low-ascending | 991 (39.45) | 960 (40.99) | 31 (18.24) |  |
| Moderate-ascending | 1245 (49.56) | 1149 (49.06) | 96 (56.47) |  |
| High-ascending | 276 (10.99) | 233 (9.95) | 43 (25.29) |  |
| BRI |  |  |  | 0.251 |
| Low-stable | 1126 (44.82) | 1054 (45.00) | 72 (42.35) |  |
| Moderate-stable | 1035 (41.20) | 968 (41.33) | 67 (39.41) |  |
| High-stable | 351 (13.97) | 320 (13.66) | 31 (18.24) |  |
| Pain |  |  |  | <0.001 |
| Low-stable | 2118 (84.32) | 2038 (87.02) | 80 (47.06) |  |
| Moderate-ascending | 253 (10.07) | 212 (9.05) | 41 (24.12) |  |
| High-ascending | 141 (5.61) | 92 (3.93) | 49 (28.82) |  |
| Sleep duration |  |  |  | <0.001 |
| High-stable | 2012 (80.10) | 1911 (81.60) | 101 (59.41) |  |
| Low-ascending | 500 (19.90) | 431 (18.40) | 69 (40.59) |  |
| Depressive symptoms |  |  |  | <0.001 |
| Low-stable | 1744 (69.43) | 1712 (73.10) | 32 (18.82) |  |
| Moderate-descending | 340 (13.54) | 293 (12.51) | 47 (27.65) |  |
| Low-ascending | 294 (11.70) | 249 (10.63) | 45 (26.47) |  |
| High-posterior-ascending | 134 (5.33) | 88 (3.76) | 46 (27.06) |  |
| Cognitive function |  |  |  | <0.001 |
| High-stable | 1648 (65.61) | 1582 (67.55) | 66 (38.82) |  |
| Low-descending | 864 (34.39) | 760 (32.45) | 104 (61.18) |  |

BMI, body mass index; ADLs, activities of daily living; BRI, body roundness index.

**Table S11.** Characteristics of participants according to BRI trajectories

| **Groups** | **Overall** | **Low-stable** | **Moderate-stable** | **High-stable** | ***P*** |
| --- | --- | --- | --- | --- | --- |
| BRI | 2512 | 1126 | 1035 | 351 |  |
| Age |  |  |  |  | 0.707 |
| 45~59 | 1157 (46.06) | 511 (45.38) | 493 (47.63) | 153 (43.59) |  |
| 60~74 | 1228 (48.89) | 558 (49.56) | 490 (47.34) | 180 (51.28) |  |
| ≥75 | 127 (5.06) | 57 (5.06) | 52 (5.02) | 18 (5.13) |  |
| Gender |  |  |  |  | <0.001 |
| Female | 1140 (45.38) | 323 (28.69) | 550 (53.14) | 267 (76.07) |  |
| Male | 1372 (54.62) | 803 (71.31) | 485 (46.86) | 84 (23.93) |  |
| Marital status |  |  |  |  | 0.249 |
| Married | 2117 (84.28) | 964 (85.61) | 862 (83.29) | 291 (82.91) |  |
| Unmarried | 395 (15.72) | 162 (14.39) | 173 (16.71) | 60 (17.09) |  |
| Residence |  |  |  |  | <0.001 |
| Rural | 1649 (65.64) | 818 (72.65) | 620 (59.90) | 211 (60.11) |  |
| Urban | 863 (34.36) | 308 (27.35) | 415 (40.10) | 140 (39.89) |  |
| Education level |  |  |  |  | 0.009 |
| Primary school or lower | 1491 (59.36) | 652 (57.90) | 606 (58.55) | 233 (66.38) |  |
| Middle school | 685 (27.27) | 312 (27.71) | 282 (27.25) | 91 (25.93) |  |
| High school or above | 336 (13.38) | 162 (14.39) | 147 (14.20) | 27 (7.69) |  |
| Drinking status |  |  |  |  | <0.001 |
| Never drinking | 1543 (61.43) | 602 (53.46) | 670 (64.73) | 271 (77.21) |  |
| Drinking ≤1/week | 448 (17.83) | 228 (20.25) | 179 (17.29) | 41 (11.68) |  |
| Drinking >1/week | 521 (20.74) | 296 (26.29) | 186 (17.97) | 39 (11.11) |  |
| Smoking status |  |  |  |  | <0.001 |
| Never smoking | 1299 (51.71) | 424 (37.66) | 616 (59.52) | 259 (73.79) |  |
| Former smoking | 427 (17.00) | 214 (19.01) | 158 (15.27) | 55 (15.67) |  |
| Current smoking | 786 (31.29) | 488 (43.34) | 261 (25.22) | 37 (10.54) |  |
| BMI |  |  |  |  | <0.001 |
| <23.9 | 1391(55.37) | 1011(89.79) | 363 (35.07) | 17 (4.84) |  |
| 24-27.9 | 809 (32.21) | 99 (8.79) | 577 (55.75) | 133 (37.89) |  |
| ≥28 | 312 (12.42) | 16 (1.42) | 95 (9.18) | 201 (57.26) |  |
| Multimorbidity status |  |  |  |  | <0.001 |
| Low-ascending | 991 (39.45) | 474 (42.10) | 405 (39.13) | 112 (31.91) |  |
| Moderate-ascending | 1245 (49.56) | 561 (49.82) | 509 (49.18) | 175 (49.86) |  |
| High-ascending | 276 (10.99) | 91 (8.08) | 121 (11.69) | 64 (18.23) |  |
| ADLs limitations |  |  |  |  | 0.251 |
| Low-stable | 2342 (93.23) | 1054 (93.61) | 968 (93.53) | 320 (91.17) |  |
| High-ascending | 170 (6.77) | 72 (6.39) | 67 (6.47) | 31 (8.83) |  |
| Pain |  |  |  |  | 0.334 |
| Low-stable | 2118 (84.32) | 949 (84.28) | 882 (85.22) | 287 (81.77) |  |
| Moderate-ascending | 253 (10.07) | 111 (9.86) | 96 (9.28) | 46 (13.11) |  |
| High-ascending | 141 (5.61) | 66 (5.86) | 57 (5.51) | 18 (5.13) |  |
| Sleep duration |  |  |  |  | 0.732 |
| High-stable | 2012 (80.10) | 902 (80.11) | 834 (80.58) | 276 (78.63) |  |
| Low-ascending | 500 (19.90) | 224 (19.89) | 201 (19.42) | 75 (21.37) |  |
| Depressive symptoms |  |  |  |  | 0.032 |
| Low-stable | 1744(69.43) | 772 (68.56) | 725 (70.05) | 247 (70.37) |  |
| Low-ascending | 294 (11.70) | 131 (11.63) | 133 (12.85) | 30 (8.55) |  |
| Moderate-descending | 340 (13.54) | 165 (14.65) | 116 (11.21) | 59 (16.81) |  |
| High-posterior-ascending | 134 (5.33) | 58 (5.15) | 61 (5.89) | 15 (4.27) |  |
| Cognitive function |  |  |  |  | 0.130 |
| High-stable | 1648 (65.61) | 730 (64.83) | 700 (67.63) | 218 (62.11) |  |
| Low-descending | 864 (34.39) | 396 (35.17) | 335 (32.37) | 133 (37.89) |  |

BMI, body mass index; ADLs, activities of daily living; BRI, body roundness index.

**Table S12.** Characteristics of participants according to pain trajectories

| **Groups** | **Overall** | **Low-stable** | **Moderate-ascending** | **High-ascending** | ***P*** |
| --- | --- | --- | --- | --- | --- |
| Pain | 2512 | 2118 | 253 | 141 |  |
| Age |  |  |  |  | 0.352 |
| 45~59 | 1157 (46.06) | 985 (46.51) | 111 (43.87) | 61 (43.26) |  |
| 60~74 | 1228 (48.89) | 1020 (48.16) | 132 (52.17) | 76 (53.90) |  |
| ≥75 | 127 (5.06) | 113 (5.34) | 10 (3.95) | 4 (2.84) |  |
| Gender |  |  |  |  | <0.001 |
| Female | 1140 (45.38) | 913 (43.11) | 144 (56.92) | 83 (58.87) |  |
| Male | 1372 (54.62) | 1205 (56.89) | 109 (43.08) | 58 (41.13) |  |
| Marital status |  |  |  |  | 0.176 |
| Married | 2117 (84.28) | 1797 (84.84) | 204 (80.63) | 116 (82.27) |  |
| Unmarried | 395 (15.72) | 321 (15.16) | 49 (19.37) | 25 (17.73) |  |
| Residence |  |  |  |  | 0.007 |
| Rural | 1649 (65.64) | 1363 (64.35) | 184 (72.73) | 102 (72.34) |  |
| Urban | 863 (34.36) | 755 (35.65) | 69 (27.27) | 39 (27.66) |  |
| Education level |  |  |  |  | <0.001 |
| Primary school or lower | 1491 (59.36) | 1201 (56.70) | 183 (72.33) | 107 (75.89) |  |
| Middle school | 685 (27.27) | 606 (28.61) | 50 (19.76) | 29 (20.57) |  |
| High school or above | 336 (13.38) | 311 (14.68) | 20 (7.91) | 5 (3.55) |  |
| Drinking status |  |  |  |  | 0.042 |
| Never drinking | 1543 (61.43) | 1281 (60.48) | 165 (65.22) | 97 (68.79) |  |
| Drinking ≤1/week | 448 (17.83) | 376 (17.75) | 50 (19.76) | 22 (15.60) |  |
| Drinking >1/week | 521 (20.74) | 461 (21.77) | 38 (15.02) | 22 (15.60) |  |
| Smoking status |  |  |  |  | 0.067 |
| Never smoking | 1299 (51.71) | 1069 (50.47) | 146 (57.71) | 84 (59.57) |  |
| Former smoking | 427 (17.00) | 372 (17.56) | 37 (14.62) | 18 (12.77) |  |
| Current smoking | 786 (31.29) | 677 (31.96) | 70 (27.67) | 39 (27.66) |  |
| BMI |  |  |  |  | 0.541 |
| <23.9 | 1391 (55.37) | 1164 (54.96) | 147 (58.10) | 80 (56.74) |  |
| 24-27.9 | 809 (32.21) | 695 (32.81) | 70 (27.67) | 44 (31.21) |  |
| ≥28 | 312 (12.42) | 259 (12.23) | 36 (14.23) | 17 (12.06) |  |
| Multimorbidity status |  |  |  |  | <0.001 |
| Low-ascending | 991 (39.45) | 916 (43.25) | 53 (20.95) | 22 (15.60) |  |
| Moderate-ascending | 1245 (49.56) | 1020 (48.16) | 141 (55.73) | 84 (59.57) |  |
| High-ascending | 276 (10.99) | 182 (8.59) | 59 (23.32) | 35 (24.82) |  |
| ADLs limitations |  |  |  |  | <0.001 |
| Low-stable | 2342 (93.23) | 2038 (96.22) | 212 (83.79) | 92 (65.25) |  |
| High-ascending | 170 (6.77) | 80 (3.78) | 41 (16.21) | 49 (34.75) |  |
| BRI |  |  |  |  | 0.334 |
| Low-stable | 1126 (44.82) | 949 (44.81) | 111 (43.87) | 66 (46.81) |  |
| Moderate-stable | 1035 (41.20) | 882 (41.64) | 96 (37.94) | 57 (40.43) |  |
| High-stable | 351 (13.97) | 287 (13.55) | 46 (18.18) | 18 (12.77) |  |
| Sleep duration |  |  |  |  | <0.001 |
| High-stable | 2012 (80.10) | 1742 (82.25) | 181 (71.54) | 89 (63.12) |  |
| Low-ascending | 500 (19.90) | 376 (17.75) | 72 (28.46) | 52 (36.88) |  |
| Depressive symptoms |  |  |  |  | <0.001 |
| Low-stable | 1744 (69.43) | 1628 (76.86) | 88 (34.78) | 28 (19.86) |  |
| Moderate-descending | 340 (13.54) | 251 (11.85) | 57 (22.53) | 32 (22.70) |  |
| Low-ascending | 294 (11.70) | 182 (8.59) | 68 (26.88) | 44 (31.21) |  |
| High-posterior-ascending | 134 (5.33) | 57 (2.69) | 40 (15.81) | 37 (26.24) |  |
| Cognitive function |  |  |  |  | <0.001 |
| High-stable | 1648 (65.61) | 1440 (67.99) | 145 (57.31) | 63 (44.68) |  |
| Low-descending | 864 (34.39) | 678 (32.01) | 108 (42.69) | 78 (55.32) |  |

BMI, body mass index; ADLs, activities of daily living; BRI, body roundness index.

**Table S13.** Characteristics of participants according to sleep duration trajectories

| **Groups** | **Overall** | **High-stable** | **Low-ascending** | ***P*** |
| --- | --- | --- | --- | --- |
| Sleep duration | 2512 | 2012 | 500 |  |
| Age |  |  |  | <0.001 |
| 45~59 | 1157 (46.06) | 965 (47.96) | 192 (38.40) |  |
| 60~74 | 1228 (48.89) | 950 (47.22) | 278 (55.60) |  |
| ≥75 | 127 (5.06) | 97 (4.82) | 30 (6.00) |  |
| Gender |  |  |  | 0.012 |
| Female | 1140 (45.38) | 888 (44.14) | 252 (50.40) |  |
| Male | 1372 (54.62) | 1124 (55.86) | 248 (49.60) |  |
| Marital status |  |  |  | 0.198 |
| Married | 2117 (84.28) | 1705 (84.74) | 412 (82.40) |  |
| Unmarried | 395 (15.72) | 307 (15.26) | 88 (17.60) |  |
| Residence |  |  |  | 0.070 |
| Rural | 1649 (65.64) | 1338 (66.50) | 311 (62.20) |  |
| Urban | 863 (34.36) | 674 (33.50) | 189 (37.80) |  |
| Education level |  |  |  | <0.001 |
| Primary school or lower | 1491 (59.36) | 1148 (57.06) | 343 (68.60) |  |
| Middle school | 685 (27.27) | 579 (28.78) | 106 (21.20) |  |
| High school or above | 336 (13.38) | 285 (14.17) | 51 (10.20) |  |
| Drinking status |  |  |  | 0.377 |
| Never drinking | 1543 (61.43) | 1231 (61.18) | 312 (62.40) |  |
| Drinking ≤1/week | 448 (17.83) | 353 (17.54) | 95 (19.00) |  |
| Drinking >1/week | 521 (20.74) | 428 (21.27) | 93 (18.60) |  |
| Smoking status |  |  |  | 0.631 |
| Never smoking | 1299 (51.71) | 1032 (51.29) | 267 (53.40) |  |
| Former smoking | 427 (17.00) | 348 (17.30) | 79 (15.80) |  |
| Current smoking | 786 (31.29) | 632 (31.41) | 154 (30.80) |  |
| BMI |  |  |  | 0.152 |
| <23.9 | 1391 (55.37) | 1098 (54.57) | 293 (58.60) |  |
| 24-27.9 | 809 (32.21) | 666 (33.10) | 143 (28.60) |  |
| ≥28 | 312 (12.42) | 248 (12.33) | 64 (12.80) |  |
| Multimorbidity status |  |  |  | <0.001 |
| Low-ascending | 991 (39.45) | 847 (42.10) | 144 (28.80) |  |
| Moderate-ascending | 1245 (49.56) | 979 (48.66) | 266 (53.20) |  |
| High-ascending | 276 (10.99) | 186 (9.24) | 90 (18.00) |  |
| ADLs limitations |  |  |  | <0.001 |
| Low-stable | 2342 (93.23) | 1911 (94.98) | 431 (86.20) |  |
| High-ascending | 170 (6.77) | 101 (5.02) | 69 (13.80) |  |
| BRI |  |  |  | 0.732 |
| Low-stable | 1126 (44.82) | 902 (44.83) | 224 (44.80) |  |
| Moderate-stable | 1035 (41.20) | 834 (41.45) | 201 (40.20) |  |
| High-stable | 351 (13.97) | 276 (13.72) | 75 (15.00) |  |
| Pain |  |  |  | <0.001 |
| Low-stable | 2118 (84.32) | 1742 (86.58) | 376 (75.20) |  |
| Moderate-ascending | 253 (10.07) | 181 (9.00) | 72 (14.40) |  |
| High-ascending | 141 (5.61) | 89 (4.42) | 52 (10.40) |  |
| Depressive symptoms |  |  |  | <0.001 |
| Low-stable | 1744 (69.43) | 1492 (74.16) | 252 (50.40) |  |
| Moderate-descending | 340 (13.54) | 215 (10.69) | 125 (25.00) |  |
| Low-ascending | 294 (11.70) | 228 (11.33) | 66 (13.20) |  |
| High-posterior-ascending | 134 (5.33) | 77 (3.83) | 57 (11.40) |  |
| Cognitive function |  |  |  | <0.001 |
| High-stable | 1648 (65.61) | 1355 (67.35) | 293 (58.60) |  |
| Low-descending | 864 (34.39) | 657 (32.65) | 207 (41.40) |  |

BMI, body mass index; ADLs, activities of daily living; BRI, body roundness index.

**Table S14.** Characteristics of participants according to depressive symptoms trajectories

| **Groups** | **Overall** | **Low-stable** | **Moderate-descending** | **Low-ascending** | **High-posterior-ascending** | ***P*** |
| --- | --- | --- | --- | --- | --- | --- |
| Depressive symptoms | 2512 | 1744 | 340 | 294 | 294 |  |
| Age |  |  |  |  |  | 0.224 |
| 45~59 | 1157 (46.06) | 822 (47.13) | 139 (40.88) | 140 (47.62) | 56 (41.79) |  |
| 60~74 | 1228 (48.89) | 835 (47.88) | 179 (52.65) | 140 (47.62) | 74 (55.22) |  |
| ≥75 | 127 (5.06) | 87 (4.99) | 22 (6.47) | 14 (4.76) | 4 (2.99) |  |
| Gender |  |  |  |  |  | <0.001 |
| Female | 1140 (45.38) | 720 (41.28) | 177 (52.06) | 154 (52.38) | 89 (66.42) |  |
| Male | 1372 (54.62) | 1024 (58.72) | 163 (47.94) | 140 (47.62) | 45 (33.58) |  |
| Marital status |  |  |  |  |  | <0.001 |
| Married | 2117 (84.28) | 1523 (87.33) | 261 (76.76) | 235 (79.93) | 98 (73.13) |  |
| Unmarried | 395 (15.72) | 221 (12.67) | 79 (23.24) | 59 (20.07) | 36 (26.87) |  |
| Residence |  |  |  |  |  | <0.001 |
| Rural | 1649 (65.64) | 1092 (62.61) | 248 (72.94) | 211 (71.77) | 98 (73.13) |  |
| Urban | 863 (34.36) | 652 (37.39) | 92 (27.06) | 83 (28.23) | 36 (26.87) |  |
| Education level |  |  |  |  |  | <0.001 |
| Primary school or lower | 1491 (59.36) | 961 (55.10) | 225 (66.18) | 200 (68.03) | 105 (78.36) |  |
| Middle school | 685 (27.27) | 502 (28.78) | 89 (26.18) | 73 (24.83) | 21 (15.67) |  |
| High school or above | 336 (13.38) | 281 (16.11) | 26 (7.65) | 21 (7.14) | 8 (5.97) |  |
| Drinking status |  |  |  |  |  | 0.002 |
| Never drinking | 1543 (61.43) | 1032 (59.17) | 223 (65.59) | 189 (64.29) | 99 (73.88) |  |
| Drinking ≤1/week | 448 (17.83) | 323 (18.52) | 64 (18.82) | 43 (14.63) | 18 (13.43) |  |
| Drinking >1/week | 521 (20.74) | 389 (22.31) | 53 (15.59) | 62 (21.09) | 17 (12.69) |  |
| Smoking status |  |  |  |  |  | <0.001 |
| Never smoking | 1299 (51.71) | 861 (49.37) | 185 (54.41) | 160 (54.42) | 93 (69.40) |  |
| Former smoking | 427 (17.00) | 323 (18.52) | 56 (16.47) | 34 (11.56) | 14 (10.45) |  |
| Current smoking | 786 (31.29) | 560 (32.11) | 99 (29.12) | 100 (34.01) | 27 (20.15) |  |
| BMI |  |  |  |  |  | 0.011 |
| <23.9 | 1391 (55.37) | 927 (53.15) | 200 (58.82) | 176 (59.86) | 88 (65.67) |  |
| 24-27.9 | 809 (32.21) | 586 (33.60) | 98 (28.82) | 86 (29.25) | 39 (29.10) |  |
| ≥28 | 312 (12.42) | 231 (13.25) | 42 (12.35) | 32 (10.88) | 7 (5.22) |  |
| Multimorbidity status |  |  |  |  |  | <0.001 |
| Low-ascending | 991 (39.45) | 776 (44.50) | 95 (27.94) | 94 (31.97) | 26 (19.40) |  |
| Moderate-ascending | 1245 (49.56) | 826 (47.36) | 192 (56.47) | 148 (50.34) | 79 (58.96) |  |
| High-ascending | 276 (10.99) | 142 (8.14) | 53 (15.59) | 52 (17.69) | 29 (21.64) |  |
| ADLs limitations |  |  |  |  |  | <0.001 |
| Low-stable | 2342 (93.23) | 1712 (98.17) | 293 (86.18) | 249 (84.69) | 88 (65.67) |  |
| High-ascending | 170 (6.77) | 32 (1.83) | 47 (13.82) | 45 (15.31) | 46 (34.33) |  |
| BRI |  |  |  |  |  | 0.032 |
| Low-stable | 1126 (44.82) | 772 (44.27) | 165 (48.53) | 131 (44.56) | 58 (43.28) |  |
| Moderate-stable | 1035 (41.20) | 725 (41.57) | 116 (34.12) | 133 (45.24) | 61 (45.52) |  |
| High-stable | 351 (13.97) | 247 (14.16) | 59 (17.35) | 30 (10.20) | 15 (11.19) |  |
| Pain |  |  |  |  |  | <0.001 |
| Low-stable | 2118 (84.32) | 1628 (93.35) | 251 (73.82) | 182 (61.90) | 57 (42.54) |  |
| Moderate-ascending | 253 (10.07) | 88 (5.05) | 57 (16.76) | 68 (23.13) | 40 (29.85) |  |
| High-ascending | 141 (5.61) | 28 (1.61) | 32 (9.41) | 44 (14.97) | 37 (27.61) |  |
| Sleep duration |  |  |  |  |  | <0.001 |
| High-stable | 2012 (80.10) | 1492 (85.55) | 215 (63.24) | 228 (77.55) | 77 (57.46) |  |
| Low-stable | 500 (19.90) | 252 (14.45) | 125 (36.76) | 66 (22.45) | 57 (42.54) |  |
| Cognitive function |  |  |  |  |  | <0.001 |
| High-stable | 1648 (65.61) | 1250 (71.67) | 183 (53.82) | 164 (55.78) | 51 (38.06) |  |
| Low-descending | 864 (34.39) | 494 (28.33) | 157 (46.18) | 130 (44.22) | 83 (61.94) |  |

BMI, body mass index; ADLs, activities of daily living; BRI, body roundness index.

**Table S15.** Characteristics of participants according to cognitive function

| **Groups** | **Overall** | **High-stable** | **Low-descending** | ***P*** |
| --- | --- | --- | --- | --- |
| Cognitive function | 2512 | 1648 | 864 |  |
| Age |  |  |  | <0.001 |
| 45~59 | 1157 (46.06) | 888 (53.88) | 269 (31.13) |  |
| 60~74 | 1228 (48.89) | 717 (43.51) | 511 (59.14) |  |
| ≥75 | 127 (5.06) | 43 (2.61) | 84 (9.72) |  |
| Gender |  |  |  | <0.001 |
| Female | 1140 (45.38) | 698 (42.35) | 442 (51.16) |  |
| Male | 1372 (54.62) | 950 (57.65) | 422 (48.84) |  |
| Marital status |  |  |  | <0.001 |
| Married | 2117 (84.28) | 1427 (86.59) | 690 (79.86) |  |
| Unmarried | 395 (15.72) | 221 (13.41) | 174 (20.14) |  |
| Residence |  |  |  | <0.001 |
| Rural | 1649 (65.64) | 1008 (61.17) | 641 (74.19) |  |
| Urban | 863 (34.36) | 640 (38.83) | 223 (25.81) |  |
| Education level |  |  |  | <0.001 |
| Primary school or lower | 1491 (59.36) | 767 (46.54) | 724 (83.80) |  |
| Middle school | 685 (27.27) | 576 (34.95) | 109 (12.62) |  |
| High school or above | 336 (13.38) | 305 (18.51) | 31 (3.59) |  |
| Drinking status |  |  |  | <0.001 |
| Never drinking | 1543 (61.43) | 960 (58.25) | 583 (67.48) |  |
| Drinking ≤1/week | 448 (17.83) | 336 (20.39) | 112 (12.96) |  |
| Drinking >1/week | 521 (20.74) | 352 (21.36) | 169 (19.56) |  |
| Smoking status |  |  |  | 0.063 |
| Never smoking | 1299 (51.71) | 825 (50.06) | 474 (54.86) |  |
| Former smoking | 427 (17.00) | 294 (17.84) | 133 (15.39) |  |
| Current smoking | 786 (31.29) | 529 (32.10) | 257 (29.75) |  |
| BMI |  |  |  | <0.001 |
| <23.9 | 1391 (55.37) | 865 (52.49) | 526 (60.88) |  |
| 24-27.9 | 809 (32.21) | 557 (33.80) | 252 (29.17) |  |
| ≥28 | 312 (12.42) | 226 (13.71) | 86 (9.95) |  |
| Multimorbidity status |  |  |  | 0.051 |
| Low-ascending | 991 (39.45) | 670 (40.66) | 321 (37.15) |  |
| Moderate-ascending | 1245 (49.56) | 813 (49.33) | 432 (50.00) |  |
| High-ascending | 276 (10.99) | 165 (10.01) | 111 (12.85) |  |
| ADLs limitations |  |  |  | <0.001 |
| Low-stable | 2342 (93.23) | 1582 (96.00) | 760 (87.96) |  |
| High-ascending | 170 (6.77) | 66 (4.00) | 104 (12.04) |  |
| BRI |  |  |  | 0.130 |
| Low-stable | 1126 (44.82) | 730 (44.30) | 396 (45.83) |  |
| Moderate-stable | 1035 (41.20) | 700 (42.48) | 335 (38.77) |  |
| High-stable | 351 (13.97) | 218 (13.23) | 133 (15.39) |  |
| Pain |  |  |  | <0.001 |
| Low-stable | 2118 (84.32) | 1440 (87.38) | 678 (78.47) |  |
| Moderate-ascending | 253 (10.07) | 145 (8.80) | 108 (12.50) |  |
| High-ascending | 141 (5.61) | 63 (3.82) | 78 (9.03) |  |
| Sleep duration |  |  |  | <0.001 |
| High-stable | 2012 (80.10) | 1355 (82.22) | 657 (76.04) |  |
| Low-ascending | 500 (19.90) | 293 (17.78) | 207 (23.96) |  |
| Depressive symptoms |  |  |  | <0.001 |
| Low-stable | 1744 (69.43) | 1250 (75.85) | 494 (57.18) |  |
| Moderate-descending | 340 (13.54) | 183 (11.10) | 157 (18.17) |  |
| Low-ascending | 294 (11.70) | 164 (9.95) | 130 (15.05) |  |
| High-posterior-ascending | 134 (5.33) | 51 (3.09) | 83 (9.61) |  |

BMI, body mass index; ADLs, activities of daily living; BRI, body roundness index.

**Table S16.** Subgroup analysis of the relationship between multimorbidity status trajectories and CVD risk

| **Subgroup** | **Overall** | **Low-ascending** | **Moderate-ascending** | **High-ascending** | **P for interaction** |
| --- | --- | --- | --- | --- | --- |
| Age |  |  |  |  | 0.604 |
| 45~59 | 1157 | Reference | 1.68 (1.09,2.59) | 3.29 (1.83,5.92) |  |
| 60~74 | 1228 | Reference | 1.44 (0.95,2.17) | 3.30 (2.09,5.21) |  |
| ≥75 | 127 | Reference | 0.14 (0.03,0.57) | 1.05 (0.21,5.24) |  |
| Gender |  |  |  |  | 0.961 |
| Female | 1140 | Reference | 1.43 (0.95,2.15) | 3.06 (1.91,4.93) |  |
| Male | 1372 | Reference | 1.39 (0.94,2.06) | 2.98 (1.86,4.79) |  |
| Marital status |  |  |  |  | 0.294 |
| Married | 2117 | Reference | 1.54 (1.13,2.09) | 3.08 (2.13,4.44) |  |
| Unmarried | 395 | Reference | 1.09 (0.51,2.33) | 3.73 (1.56,8.91) |  |
| Residence |  |  |  |  | 0.404 |
| Rural | 1649 | Reference | 1.63 (1.14,2.32) | 3.22 (2.10,4.94) |  |
| Urban | 863 | Reference | 1.18 (0.74,1.90) | 3.31 (1.91,5.76) |  |
| Education level |  |  |  |  | 0.055 |
| Primary school or lower | 1491 | Reference | 1.15 (0.81,1.64) | 3.14 (2.11,4.65) |  |
| Middle school | 685 | Reference | 1.67 (0.91,3.06) | 2.65 (1.08,6.48) |  |
| High school or above | 336 | Reference | 2.92 (1.31,6.51) | 2.27 (0.75,6.84) |  |
| Drinking status |  |  |  |  | 0.230 |
| Never drinking | 1543 | Reference | 1.63 (1.13,2.34) | 3.62 (2.38,5.49) |  |
| Drinking ≤1/week | 448 | Reference | 0.97 (0.52,1.80) | 1.65 (0.58,4.73) |  |
| Drinking >1/week | 521 | Reference | 1.36 (0.67,2.76) | 3.95 (1.75,8.92) |  |
| Smoking status |  |  |  |  | 0.717 |
| Never smoking | 1299 | Reference | 1.40 (0.95,2.06) | 3.20 (2.03,5.03) |  |
| Former smoking | 427 | Reference | 1.54 (0.80,2.96) | 4.33 (2.05,9.18) |  |
| Current smoking | 786 | Reference | 1.65 (0.96,2.83) | 2.40 (1.16,4.97) |  |
| BMI |  |  |  |  | 0.985 |
| <23.9 | 1391 | Reference | 1.39 (0.93,2.06) | 3.17 (1.94,5.19) |  |
| 24-27.9 | 809 | Reference | 1.48 (0.91,2.40) | 2.92 (1.63,5.21) |  |
| ≥28 | 312 | Reference | 1.58 (0.72,3.45) | 3.42 (1.45,8.09) |  |
| SBP |  |  |  |  | 0.393 |
| ≤118.5 | 858 | Reference | 1.39 (0.82,2.37) | 3.10 (1.56,6.13) |  |
| 118.5-136 | 873 | Reference | 2.20 (1.26,3.83) | 4.52 (2.25,9.08) |  |
| ≥136 | 781 | Reference | 1.08 (0.71,1.66) | 2.65 (1.65,4.27) |  |
| DBP |  |  |  |  | 0.713 |
| ≤70 | 818 | Reference | 1.57 (0.93,2.64) | 4.27 (2.28,7.99) |  |
| 70-80 | 896 | Reference | 1.60 (0.94,2.73) | 3.16 (1.64,6.09) |  |
| ≥80 | 798 | Reference | 1.21 (0.78,1.88) | 2.71 (1.63,4.50) |  |

In addition to the stratification variables themselves, triglycerides, creatinine, high-density lipoprotein cholesterol, low-density lipoprotein cholesterol, total cholesterol, fasting blood glucose, uric acid, and C-reactive protein were adjusted. CVD, cardiovascular disease; BMI, body mass index; SBP, systolic blood pressure; DBP, diastolic blood pressure.

**Table S17.** Subgroup analysis of the relationship between ADLs limitations trajectories and CVD risk

| **Subgroup** | **Overall** | **Low-stable** | **High-ascending** | **P for interaction** |
| --- | --- | --- | --- | --- |
| Age |  |  |  | 0.881 |
| 45~59 | 1157 | Reference | 3.10 (1.61,5.98) |  |
| 60~74 | 1228 | Reference | 2.80 (1.85,4.22) |  |
| ≥75 | 127 | Reference | 3.87 (1.11,13.49) |  |
| Gender |  |  |  | 0.539 |
| Female | 1140 | Reference | 2.41 (1.55,3.76) |  |
| Male | 1372 | Reference | 2.97 (1.82,4.85) |  |
| Marital status |  |  |  | 0.862 |
| Married | 2117 | Reference | 2.62 (1.85,3.72) |  |
| Unmarried | 395 | Reference | 3.48 (1.36,8.93) |  |
| Residence |  |  |  | 0.877 |
| Rural | 1649 | Reference | 2.67 (1.83,3.89) |  |
| Urban | 863 | Reference | 2.55 (1.32,4.93) |  |
| Education level |  |  |  | 0.993 |
| Primary school or lower | 1491 | Reference | 2.75 (1.91,3.95) |  |
| Middle school | 685 | Reference | 3.48 (1.42,8.54) |  |
| High school or above | 336 | Reference | 1.52 (0.21,10.87) |  |
| Drinking status |  |  |  | 0.548 |
| Never drinking | 1543 | Reference | 2.65 (1.83,3.85) |  |
| Drinking ≤1/week | 448 | Reference | 5.58 (1.95,15.91) |  |
| Drinking >1/week | 521 | Reference | 2.76 (0.93,8.16) |  |
| Smoking status |  |  |  | 0.654 |
| Never smoking | 1299 | Reference | 2.28 (1.45,3.58) |  |
| Former smoking | 427 | Reference | 2.49 (1.20,5.19) |  |
| Current smoking | 786 | Reference | 4.55 (2.26,9.17) |  |
| BMI |  |  |  | 0.529 |
| <23.9 | 1391 | Reference | 2.42 (1.52,3.87) |  |
| 24-27.9 | 809 | Reference | 3.04 (1.72,5.37) |  |
| ≥28 | 312 | Reference | 2.80 (1.15,6.79) |  |
| SBP |  |  |  | 0.162 |
| ≤118.5 | 858 | Reference | 1.55 (0.67,3.61) |  |
| 118.5-136 | 873 | Reference | 3.18 (1.67,6.07) |  |
| ≥136 | 781 | Reference | 3.23 (2.08,5.01) |  |
| DBP |  |  |  | 0.003 |
| ≤70 | 818 | Reference | 0.93 (0.39,2.24) |  |
| 70-80 | 896 | Reference | 2.97 (1.64,5.38) |  |
| ≥80 | 798 | Reference | 4.27 (2.68,6.79) |  |

In addition to the stratification variables themselves, triglycerides, creatinine, high-density lipoprotein cholesterol, low-density lipoprotein cholesterol, total cholesterol, fasting blood glucose, uric acid, and C-reactive protein were adjusted. ADLs, activities of daily living; CVD, cardiovascular disease; BMI, body mass index; SBP, systolic blood pressure; DBP, diastolic blood pressure.

**Table S18.** Subgroup analysis of the relationship between BRI trajectories and CVD risk

| **Subgroup** | **Overall** | **Low-stable** | **Moderate-stable** | **High-stable** | **P for interaction** |
| --- | --- | --- | --- | --- | --- |
| Age |  |  |  |  | 0.533 |
| 45~59 | 1157 | Reference | 1.50 (0.88, 2.56) | 1.77 (0.78, 4.01) |  |
| 60~74 | 1228 | Reference | 1.34 (0.85, 2.13) | 1.88 (0.96, 3.67) |  |
| ≥75 | 127 | Reference | 0.50 (0.13, 1.96) | 0.62 (0.09, 4.08) |  |
| Gender |  |  |  |  | 0.880 |
| Female | 1140 | Reference | 1.34 (0.79, 2.26) | 1.61 (0.82, 3.17) |  |
| Male | 1372 | Reference | 1.34 (0.86, 2.11) | 1.92 (0.89, 4.14) |  |
| Marital status |  |  |  |  | 0.165 |
| Married | 2117 | Reference | 1.26 (0.87, 1.82) | 1.64 (0.97, 2.77) |  |
| Unmarried | 395 | Reference | 1.75 (0.77, 3.99) | 2.13 (0.55, 8.23) |  |
| Residence |  |  |  |  | 0.840 |
| Rural | 1649 | Reference | 1.41 (0.94, 2.11) | 1.92 (1.05, 3.50) |  |
| Urban | 863 | Reference | 1.15 (0.64, 2.05) | 1.20 (0.52, 2.76) |  |
| Education level |  |  |  |  | 0.449 |
| Primary school or lower | 1491 | Reference | 1.39 (0.91, 2.12) | 1.96 (1.08, 3.56) |  |
| Middle school | 685 | Reference | 1.46 (0.69, 3.06) | 1.69 (0.52, 5.47) |  |
| High school or above | 336 | Reference | 1.60 (0.66, 3.90) | 0.71 (0.14, 3.57) |  |
| Drinking status |  |  |  |  | 0.583 |
| Drinking >1/week | 521 | Reference | 0.94 (0.44, 2.02) | 1.70 (0.46, 6.34) |  |
| Drinking ≤1/week | 448 | Reference | 2.24 (0.99, 5.10) | 1.45 (0.34, 6.23) |  |
| Never drinking | 1543 | Reference | 1.34 (0.88, 2.05) | 1.82 (1.01, 3.29) |  |
| Smoking status |  |  |  |  | 0.947 |
| Never smoking | 1299 | Reference | 1.29 (0.80, 2.07) | 1.40 (0.74, 2.67) |  |
| Former smoking | 427 | Reference | 1.20 (0.57, 2.50) | 1.88 (0.62, 5.74) |  |
| Current smoking | 786 | Reference | 1.79 (0.96, 3.34) | 2.07 (0.62, 6.93) |  |
| BMI |  |  |  |  | 0.714 |
| <23.9 | 1391 | Reference | 1.36 (0.89,2.07) | 0.73 (0.17,3.17) |  |
| 24-27.9 | 809 | Reference | 1.37 (0.63,2.95) | 2.40 (0.96,5.99) |  |
| ≥28 | 312 | Reference | 5.72 (0.62,52.67) | 6.28 (0.58,67.48) |  |
| SBP |  |  |  |  | 0.769 |
| ≤118.5 | 858 | Reference | 0.98 (0.50,1.94) | 1.25 (0.45,3.52) |  |
| 118.5-136 | 873 | Reference | 1.65 (0.88,3.11) | 1.54 (0.62,3.86) |  |
| ≥136 | 781 | Reference | 1.26 (0.77,2.07) | 2.31 (1.15,4.64) |  |
| DBP |  |  |  |  | 0.777 |
| ≤70 | 818 | Reference | 0.94 (0.52,1.70) | 0.71 (0.27,1.90) |  |
| 70-80 | 896 | Reference | 1.05 (0.54,2.02) | 1.31 (0.52,3.34) |  |
| ≥80 | 798 | Reference | 1.95 (1.14,3.32) | 3.61 (1.73,7.54) |  |

In addition to the stratification variables themselves, triglycerides, creatinine, high-density lipoprotein cholesterol, low-density lipoprotein cholesterol, total cholesterol, fasting blood glucose, uric acid, and C-reactive protein were adjusted. BRI, body roundness index; CVD, cardiovascular disease; BMI, body mass index; SBP, systolic blood pressure; DBP, diastolic blood pressure.

**Table S19.** Subgroup analysis of the relationship between pain trajectories and CVD risk

| **Subgroup** | **Overall** | **Low-stable** | **Moderate-ascending** | **High-ascending** | **P for interaction** |
| --- | --- | --- | --- | --- | --- |
| Age |  |  |  |  | 0.497 |
| 45~59 | 1157 | Reference | 1.12 (0.59, 2.14) | 2.41 (1.26, 4.61) |  |
| 60~74 | 1228 | Reference | 1.85 (1.21, 2.85) | 2.10 (1.25, 3.54) |  |
| ≥75 | 127 | Reference | 4.58 (0.78, 26.8) | 34.86 (3.27, 371.18) |  |
| Gender |  |  |  |  | 0.138 |
| Female | 1140 | Reference | 1.20 (0.73, 1.95) | 1.96 (1.18, 3.27) |  |
| Male | 1372 | Reference | 2.09 (1.28, 3.41) | 3.18 (1.74, 5.80) |  |
| Marital status |  |  |  |  | 0.907 |
| Married | 2117 | Reference | 1.53 (1.05, 2.23) | 2.23 (1.45, 3.42) |  |
| Unmarried | 395 | Reference | 2.30 (0.92, 5.74) | 3.99 (1.44, 11.01) |  |
| Residence |  |  |  |  | 0.300 |
| Rural | 1649 | Reference | 1.52 (1.01, 2.29) | 1.96 (1.20, 3.20) |  |
| Urban | 863 | Reference | 1.50 (0.77, 2.89) | 3.29 (1.76, 6.15) |  |
| Education level |  |  |  |  | 0.616 |
| Primary school or lower | 1491 | Reference | 1.55 (1.03, 2.32) | 2.61 (1.71, 3.98) |  |
| Middle school | 685 | Reference | 1.74 (0.79, 3.82) | 1.20 (0.34, 4.22) |  |
| High school or above | 336 | Reference | 0.51 (0.09, 2.90) | 1.62 (0.2, 13.33) |  |
| Drinking status |  |  |  |  | 0.805 |
| Never drinking | 1543 | Reference | 1.54 (1.02, 2.33) | 2.04 (1.28, 3.25) |  |
| Drinking ≤1/week | 448 | Reference | 1.53 (0.65, 3.62) | 4.74 (1.76, 12.77) |  |
| Drinking >1/week | 521 | Reference | 1.69 (0.62, 4.62) | 2.48 (0.78, 7.89) |  |
| Smoking status |  |  |  |  | 0.010 |
| Never smoking | 1299 | Reference | 1.37 (0.86, 2.17) | 1.40 (0.79, 2.46) |  |
| Former smoking | 427 | Reference | 2.89 (1.40, 5.96) | 3.33 (1.28, 8.68) |  |
| Current smoking | 786 | Reference | 1.16 (0.49, 2.75) | 5.56 (2.69, 11.51) |  |
| BMI |  |  |  |  | 0.996 |
| <23.9 | 1391 | Reference | 1.62 (0.99,2.64) | 2.69 (1.54,4.68) |  |
| 24-27.9 | 809 | Reference | 1.65 (0.88,3.10) | 2.32 (1.17,4.60) |  |
| ≥28 | 312 | Reference | 1.31 (0.57,3.04) | 2.54 (0.98,6.61) |  |
| SBP |  |  |  |  | 0.550 |
| ≤118.5 | 858 | Reference | 1.26 (0.56,2.83) | 1.75 (0.80,3.81) |  |
| 118.5-136 | 873 | Reference | 1.13 (0.57,2.26) | 2.52 (1.19,5.30) |  |
| ≥136 | 781 | Reference | 1.95 (1.22,3.11) | 2.79 (1.55,5.00) |  |
| DBP |  |  |  |  | 0.700 |
| ≤70 | 818 | Reference | 1.70 (0.89,3.24) | 1.70 (0.79,3.67) |  |
| 70-80 | 896 | Reference | 0.88 (0.41,1.89) | 2.19 (1.09,4.40) |  |
| ≥80 | 798 | Reference | 1.94 (1.17,3.20) | 2.80 (1.52,5.17) |  |

In addition to the stratification variables themselves, triglycerides, creatinine, high-density lipoprotein cholesterol, low-density lipoprotein cholesterol, total cholesterol, fasting blood glucose, uric acid, and C-reactive protein were adjusted. CVD, cardiovascular disease; BMI, body mass index; SBP, systolic blood pressure; DBP, diastolic blood pressure.

**Table S20.** Subgroup analysis of the relationship between sleep duration trajectories and CVD risk

| **Groups** | **Overall** | **High-stable** | **Low-ascending** | **P for interaction** |
| --- | --- | --- | --- | --- |
| Age |  |  |  | 0.929 |
| 45~59 | 1157 | Reference | 1.26 (0.78,2.05) |  |
| 60~74 | 1228 | Reference | 1.36 (0.96,1.93) |  |
| ≥75 | 127 | Reference | 1.56 (0.51,4.75) |  |
| Gender |  |  |  | 0.919 |
| Female | 1140 | Reference | 1.32 (0.91,1.91) |  |
| Male | 1372 | Reference | 1.35 (0.90,2.03) |  |
| Marital status |  |  |  | 0.044 |
| Married | 2117 | Reference | 1.15 (0.85,1.56) |  |
| Unmarried | 395 | Reference | 2.68 (1.38,5.23) |  |
| Residence |  |  |  | 0.063 |
| Rural | 1649 | Reference | 1.69 (1.22,2.35) |  |
| Urban | 863 | Reference | 0.92 (0.57,1.50) |  |
| Education level |  |  |  | 0.389 |
| Primary school or lower | 1491 | Reference | 1.41 (1.03,1.94) |  |
| Middle school | 685 | Reference | 1.35 (0.71,2.59) |  |
| High school or above | 336 | Reference | 0.73 (0.27,2.00) |  |
| Drinking status |  |  |  | 0.184 |
| Never drinking | 1543 | Reference | 1.60 (1.16,2.21) |  |
| Drinking ≤1/week | 448 | Reference | 0.77 (0.36,1.64) |  |
| Drinking >1/week | 521 | Reference | 1.04 (0.48,2.24) |  |
| Smoking status |  |  |  | 0.075 |
| Never smoking | 1299 | Reference | 1.03 (0.71,1.51) |  |
| Former smoking | 427 | Reference | 2.82 (1.56,5.10) |  |
| Current smoking | 786 | Reference | 1.60 (0.91,2.82) |  |
| BMI |  |  |  | 0.098 |
| <23.9 | 1391 | Reference | 1.77 (1.22,2.56) |  |
| 24-27.9 | 809 | Reference | 1.23 (0.75,2.01) |  |
| ≥28 | 312 | Reference | 0.62 (0.29,1.32) |  |
| SBP |  |  |  | 0.165 |
| ≤118.5 | 858 | Reference | 1.79 (1.09,2.96) |  |
| 118.5-136 | 873 | Reference | 1.73 (1.05,2.84) |  |
| ≥136 | 781 | Reference | 0.97 (0.63,1.50) |  |
| DBP |  |  |  | 0.099 |
| ≤70 | 818 | Reference | 1.78 (1.11,2.85) |  |
| 70-80 | 896 | Reference | 1.87 (1.14,3.09) |  |
| ≥80 | 798 | Reference | 0.94 (0.59,1.49) |  |

In addition to the stratification variables themselves, triglycerides, creatinine, high-density lipoprotein cholesterol, low-density lipoprotein cholesterol, total cholesterol, fasting blood glucose, uric acid, and C-reactive protein were adjusted. CVD, cardiovascular disease; BMI, body mass index; SBP, systolic blood pressure; DBP, diastolic blood pressure.

**Table S21.** Subgroup analysis of the relationship between depressive symptoms trajectories and CVD risk

| **Groups** | **Overall** | **Low-stable** | **Moderate-descending** | **Low-ascending** | **High-posterior-ascending** | **P for interaction** |
| --- | --- | --- | --- | --- | --- | --- |
| Age |  |  |  |  |  | 0.165 |
| 45~59 | 1157 | Reference | 1.52 (0.85, 2.73) | 2.40 (1.45, 3.98) | 2.32 (1.08, 4.98) |  |
| 60~74 | 1228 | Reference | 1.46 (0.94, 2.27) | 1.47 (0.92, 2.37) | 2.95 (1.76, 4.94) |  |
| ≥75 | 127 | Reference | 1.97 (0.51, 7.66) | 0.41 (0.04, 4.24) | 3.38 (0.28, 40.59) |  |
| Gender |  |  |  |  |  | 0.027 |
| Female | 1140 | Reference | 2.33 (1.12, 4.85) | 1.87 (1.18, 2.95) | 2.93 (1.75, 4.92) |  |
| Male | 1372 | Reference | 2.43 (1.56, 3.77) | 1.64 (0.99, 2.72) | 2.33 (1.12, 4.85) |  |
| Marital status |  |  |  |  |  | 0.400 |
| Married | 2117 | Reference | 1.45 (1.00, 2.10) | 1.92 (1.34, 2.74) | 2.56 (1.63, 4.02) |  |
| Unmarried | 395 | Reference | 1.62 (0.73, 3.59) | 1.06 (0.38, 2.95) | 2.40 (0.89, 6.47) |  |
| Residence |  |  |  |  |  | 0.243 |
| Rural | 1649 | Reference | 1.55 (1.04, 2.30) | 1.42 (0.92, 2.19) | 2.91 (1.80, 4.71) |  |
| Urban | 863 | Reference | 1.74 (0.95, 3.19) | 2.66 (1.54, 4.59) | 2.08 (0.92, 4.67) |  |
| Education level |  |  |  |  |  | 0.286 |
| Primary school or lower | 1491 | Reference | 1.40 (0.93, 2.09) | 1.47 (0.96, 2.24) | 3.07 (1.95, 4.83) |  |
| Middle school | 685 | Reference | 2.22 (1.08, 4.57) | 2.40 (1.17, 4.92) | 1.47 (0.34, 6.45) |  |
| High school or above | 336 | Reference | 1.42 (0.41, 4.97) | 2.12 (0.75, 6.05) | 0.85 (0.09, 8.06) |  |
| Drinking status |  |  |  |  |  | 0.986 |
| Never drinking | 1543 | Reference | 1.63 (1.10, 2.41) | 1.89 (1.25, 2.85) | 2.62 (1.61, 4.27) |  |
| Drinking ≤1/week | 448 | Reference | 1.60 (0.69, 3.73) | 1.64 (0.67, 3.99) | 3.53 (1.03, 12.12) |  |
| Drinking >1/week | 521 | Reference | 1.13 (0.42, 3.07) | 1.41 (0.58, 3.41) | 3.78 (1.24, 11.54) |  |
| Smoking status |  |  |  |  |  | 0.198 |
| Never smoking | 1299 | Reference | 1.01 (0.62, 1.66) | 1.81 (1.16, 2.82) | 2.35 (1.41, 3.91) |  |
| Former smoking | 427 | Reference | 2.57 (1.25, 5.27) | 1.30 (0.51, 3.29) | 4.17 (1.30, 13.40) |  |
| Current smoking | 786 | Reference | 2.91 (1.55, 5.47) | 2.20 (1.11, 4.37) | 2.80 (1.05, 7.42) |  |
| BMI |  |  |  |  |  | 0.418 |
| <23.9 | 1391 | Reference | 1.56 (0.96,2.52) | 1.74 (1.07,2.84) | 3.70 (2.20,6.23) |  |
| 24-27.9 | 809 | Reference | 1.91 (1.09,3.34) | 1.77 (0.97,3.24) | 1.41 (0.59,3.38) |  |
| ≥28 | 312 | Reference | 0.92 (0.38,2.25) | 2.10 (0.96,4.61) | 3.88 (1.03,14.56) |  |
| SBP |  |  |  |  |  | 0.713 |
| ≤118.5 | 858 | Reference | 1.53 (0.76,3.09) | 1.73 (0.86,3.50) | 3.27 (1.57,6.79) |  |
| 118.5-136 | 873 | Reference | 2.18 (1.24,3.81) | 1.89 (1.01,3.51) | 1.78 (0.61,5.24) |  |
| ≥136 | 781 | Reference | 1.18 (0.69,2.02) | 1.70 (1.02,2.82) | 2.89 (1.60,5.21) |  |
| DBP |  |  |  |  |  | 0.817 |
| ≤70 | 818 | Reference | 1.86 (1.02,3.41) | 1.88 (1.02,3.47) | 2.96 (1.46,6.00) |  |
| 70-80 | 896 | Reference | 1.41 (0.75,2.63) | 0.94 (0.44,2.04) | 2.28 (1.07,4.85) |  |
| ≥80 | 798 | Reference | 1.58 (0.93,2.68) | 2.08 (1.26,3.44) | 2.42 (1.18,4.96) |  |

In addition to the stratification variables themselves, triglycerides, creatinine, high-density lipoprotein cholesterol, low-density lipoprotein cholesterol, total cholesterol, fasting blood glucose, uric acid, and C-reactive protein were adjusted. CVD, cardiovascular disease; BMI, body mass index; SBP, systolic blood pressure; DBP, diastolic blood pressure.

**Table S22.** Subgroup analysis of the relationship between cognitive function trajectories and CVD risk

| **Groups** | **Overall** | **High-stable** | **Low-descending** | **P for interaction** |
| --- | --- | --- | --- | --- |
| Age |  |  |  | 0.536 |
| 45~59 | 1157 | Reference | 1.17 (0.74,1.85) |  |
| 60~74 | 1228 | Reference | 1.04 (0.74,1.46) |  |
| ≥75 | 127 | Reference | 10.71 (2.33,49.34) |  |
| Gender |  |  |  | 0.585 |
| Female | 1140 | Reference | 1.12 (0.77,1.63) |  |
| Male | 1372 | Reference | 1.25 (0.87,1.81) |  |
| Marital status |  |  |  | 0.912 |
| Married | 2117 | Reference | 1.17 (0.88,1.54) |  |
| Unmarried | 395 | Reference | 1.33 (0.65,2.70) |  |
| Residence |  |  |  | 0.523 |
| Rural | 1649 | Reference | 1.17 (0.85,1.61) |  |
| Urban | 863 | Reference | 1.27 (0.80,2.03) |  |
| Education level |  |  |  | 0.523 |
| Primary school or lower | 1491 | Reference | 1.20 (0.89,1.62) |  |
| Middle school | 685 | Reference | 0.96 (0.46,2.01) |  |
| High school or above | 336 | Reference | 2.06 (0.85,4.99) |  |
| Drinking status |  |  |  | 0.498 |
| Never drinking | 1543 | Reference | 1.23 (0.89,1.69) |  |
| Drinking ≤1/week | 448 | Reference | 1.33 (0.67,2.61) |  |
| Drinking >1/week | 521 | Reference | 1.10 (0.56,2.15) |  |
| Smoking status |  |  |  | 0.526 |
| Never smoking | 1299 | Reference | 1.07 (0.75,1.53) |  |
| Former smoking | 427 | Reference | 1.23 (0.70,2.17) |  |
| Current smoking | 786 | Reference | 1.45 (0.84,2.49) |  |
| BMI |  |  |  | 0.895 |
| <23.9 | 1391 | Reference | 1.25 (0.86,1.81) |  |
| 24-27.9 | 809 | Reference | 1.39 (0.88,2.17) |  |
| ≥28 | 312 | Reference | 0.68 (0.35,1.32) |  |
| SBP |  |  |  | 0.472 |
| ≤118.5 | 858 | Reference | 1.25 (0.71,2.17) |  |
| 118.5-136 | 873 | Reference | 1.05 (0.65,1.69) |  |
| ≥136 | 781 | Reference | 1.37 (0.93,2.01) |  |
| DBP |  |  |  | 0.179 |
| ≤70 | 818 | Reference | 0.95 (0.58,1.58) |  |
| 70-80 | 896 | Reference | 1.37 (0.82,2.28) |  |
| ≥80 | 798 | Reference | 1.34 (0.90,2.00) |  |

In addition to the stratification variables themselves, triglycerides, creatinine, high-density lipoprotein cholesterol, low-density lipoprotein cholesterol, total cholesterol, fasting blood glucose, uric acid, and C-reactive protein were adjusted. CVD, cardiovascular disease; BMI, body mass index; SBP, systolic blood pressure; DBP, diastolic blood pressure.

**Table S23.** Classification of variables

| \| **Category** \| \| --- \| | \| **Variables** \| \| --- \| |
| --- | --- | --- | --- |
| Sociodemographic variables | Age, Gender (Female/Male), Marital status (Married/Unmarried), Residence (Rural/Urban), Education level (Primary school or lower/Middle school/High school or above), Drinking status (Never drinking/Drinking ≤1/week/Drinking >1/week), Smoking status (Never smoking/Former smoking/Current smoking) |
| physical examination variables | BMI (kg/m²), SBP (mmHg), DBP (mmHg), Pulse (beats per minute), Peak expiratory flow (L/min), Handgrip strength (kg), Walking speed (m/s), Balance ability (Impaired/Normal) |
| Blood test variables | WBC (×10⁹/L), HGB (g/L), HCT (%), MCV (fL), PLT (×10⁹/L), TG (mmol/L), CREA (μmol/L), BUN (mmol/L), HDL-C (mmol/L), LDL-C (mmol/L), TC (mmol/L), GLU (mmol/L), UA (μmol/L), CysC (mg/L), CRP (mg/L), HbA1c (% or mmol/mol) |
| Trajectories of health conditions variables | multimorbidity status (low-ascending/moderate-ascending/high-ascending), ADLs limitations (low-stable/high-ascending), BRI (low-stable/moderate-stable/high-stable), pain (low-stable/moderate-ascending/high-ascending), sleep duration (high-stable/low-ascending), depressive symptoms (low-stable/moderate-descending/low-ascending/high-posterior-ascending), cognitive function (high-stable/low-descending) |

BMI, body mass index; SBP, systolic blood pressure; DBP, diastolic blood pressure; WBC, white blood cell count; HGB, hemoglobin; HCT, hematocrit; MCV, mean corpuscular volume; PLT, platelet count; TG, triglycerides; CREA, creatinine; BUN, blood urea nitrogen; HDL-C, high-density lipoprotein cholesterol; LDL-C, low-density lipoprotein cholesterol; TC, total cholesterol; GLU, glucose; UA, uric acid; CYSC, cystatin c; CRP, c-reactive protein; HBALC, hemoglobin a1c; BRI, body roundness index; ADLs, activities of daily living; CVD, cardiovascular disease.

**Table S24.** AUC of ten ML algorithms across different variable groups

| **Model** | **All variables** | **Exclude trajectories of health conditions variables** | **Exclude physical examination variables** | **Exclude sociodemographic variables** | **Exclude blood test variables** |
| --- | --- | --- | --- | --- | --- |
| LR | 0.718 (0.655 - 0.781) | 0.635 (0.558 - 0.712) | 0.705 (0.636 - 0.773) | 0.716 (0.652 - 0.779) | 0.732 (0.673 - 0.791) |
| SVM | 0.681 (0.613 - 0.749) | 0.590 (0.513 - 0.667) | 0.651 (0.581 - 0.721) | 0.672 (0.604 - 0.740) | 0.699 (0.640 - 0.758) |
| GBM | 0.721 (0.655 - 0.787) | 0.643 (0.566 - 0.720) | 0.693 (0.619 - 0.766) | 0.722 (0.656 - 0.787) | 0.738 (0.678 - 0.798) |
| NN | 0.605 (0.526 - 0.685) | 0.550 (0.472 - 0.627) | 0.649 (0.579 - 0.718) | 0.657 (0.587 - 0.727) | 0.686 (0.619 - 0.752) |
| RF | 0.702 (0.633 - 0.772) | 0.626 (0.544 - 0.708) | 0.679 (0.602 - 0.756) | 0.698 (0.627 - 0.768) | 0.737 (0.674 - 0.800) |
| XGBoost | 0.706 (0.634 - 0.778) | 0.639 (0.566 - 0.712) | 0.706 (0.632 - 0.779) | 0.707 (0.633 - 0.780) | 0.726 (0.665 - 0.786) |
| KNN | 0.624 (0.546 - 0.701) | 0.523 (0.440 - 0.606) | 0.616 (0.537 - 0.695) | 0.585 (0.509 - 0.660) | 0.665 (0.592 - 0.739) |
| AdaBoost | 0.707 (0.637 - 0.777) | 0.651 (0.574 - 0.728) | 0.665 (0.586 - 0.745) | 0.707 (0.637 - 0.777) | 0.690 (0.619 - 0.761) |
| LightGBM | 0.623 (0.541 - 0.705) | 0.572 (0.488 - 0.655) | 0.617 (0.534 - 0.700) | 0.618 (0.537 - 0.700) | 0.627 (0.547 - 0.707) |
| CatBoost | 0.688 (0.618 - 0.758) | 0.641 (0.571 - 0.710) | 0.710 (0.640 - 0.781) | 0.718 (0.651 - 0.785) | 0.712 (0.648 - 0.776) |

AUC, area under the receiver operating characteristic curve; ML, machine learning; LR, logistic regression; SVM, support vector machine; GBM, gradient boosting machine; NN, neural network; RF, random forest; XGBoost, extreme gradient boosting; KNN, k-nearest neighbors; AdaBoost, adaptive boosting; LightGBM, light gradient boosting machine; CatBoost, categorical boosting.

**
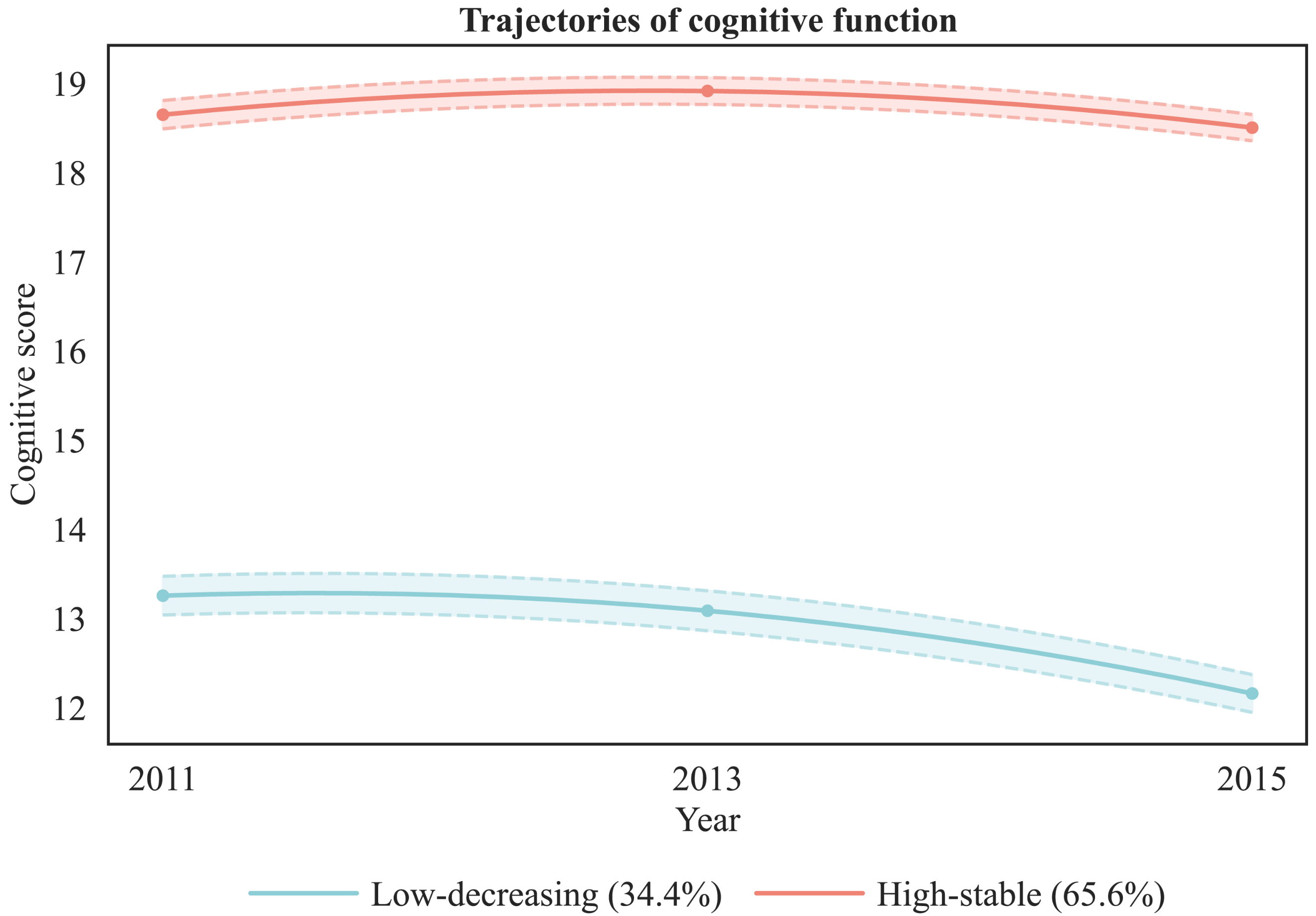
**

Fig. S1. Trajectories of cognitive function. Low-decreasing (blue line, lower baseline cognitive scores with a progressive decline over time; 34.4% of participants) and High-stable (red line, high baseline cognitive scores with stability maintained throughout the study period; 65.6% of participants).
